# MxB binds to the HIV-1 core and prevents the uncoating process of HIV-1

**DOI:** 10.1186/s12977-014-0068-x

**Published:** 2014-08-14

**Authors:** Thomas Fricke, Tommy E White, Bianca Schulte, Daniel A de Souza Aranha Vieira, Adarsh Dharan, Edward M Campbell, Alberto Brandariz-Nuñez, Felipe Diaz-Griffero

**Affiliations:** Department of Microbiology and Immunology, Albert Einstein College of Medicine, Bronx, NY 10461 USA; Department of Microbiology and Immunology, Loyola University, Chicago, IL 60153 USA; Albert Einstein College of Medicine, 1301 Morris Park – Price Center 501, Bronx, NY 10461 USA

**Keywords:** MxB, HIV-1, Uncoating, Capsid, Core, IFN-α, Binding, Oligomerization

## Abstract

**Background:**

The IFN-α-inducible restriction factor MxB blocks HIV-1 infection after reverse transcription but prior to integration. Genetic evidence suggested that capsid is the viral determinant for restriction by MxB. This work explores the ability of MxB to bind to the HIV-1 core, and the role of capsid-binding in restriction.

**Results:**

We showed that MxB binds to the HIV-1 core and that this interaction leads to inhibition of the uncoating process of HIV-1. These results identify MxB as an endogenously expressed protein with the ability to inhibit HIV-1 uncoating. In addition, we found that a benzimidazole-based compound known to have a binding pocket on the surface of the HIV-1 capsid prevents the binding of MxB to capsid. The use of this small-molecule identified the MxB binding region on the surface of the HIV-1 core. Domain mapping experiments revealed the following requirements for restriction: 1) MxB binding to the HIV-1 capsid, which requires the 20 N-terminal amino acids, and 2) oligomerization of MxB, which is mediated by the C-terminal domain provides the avidity for the interaction of MxB with the HIV-1 core.

**Conclusions:**

Overall our work establishes that MxB binds to the HIV-1 core and inhibits the uncoating process of HIV-1. Moreover, we demonstrated that HIV-1 restriction by MxB requires capsid binding and oligomerization.

**Electronic supplementary material:**

The online version of this article (doi:10.1186/s12977-014-0068-x) contains supplementary material, which is available to authorized users.

## Background

The myxovirus resistance proteins (Mxs) represent a family of interferon-inducible factors with a wide range of antiviral activities [1,2]. The MxB gene was originally cloned from a human glioblastoma cell line treated with interferon-α (IFN-α) [3,4]. MxB as well as the related protein MxA belongs to the dynamin-like family of proteins, which have diverse functions ranging from vesicle transport to antiviral activity [1,5-10]. The most studied dynamin-like protein that exhibits antiviral activity is MxA [1,2]. Contrary to MxB, the antiviral role of MxA has been extensively studied for viruses including influenza [1,11-14], tick-born Thogoto [15], African swine fever [16], hepatitis B [17], and La Crosse [18,19]. Only recently the antiviral activity of the long form of MxB was described [8,20-22]; these investigations lead to the discovery that the IFN-α-inducible protein MxB blocks HIV-1 infection.

Genetic evidence suggested that HIV-1 capsid is the determinant for the ability of MxB to block HIV-1 infection [8,21,22]. HIV-1 viruses bearing capsid changes such as P90A, G89V and N57S escape MxB restriction suggesting that capsid is the determinant for the block imposed by MxB. These experiments imply that MxB is directly interacting with the HIV-1 core early during infection. However, the ability of MxB to associate with HIV-1 cores has not been explored.

MxB blocks HIV-1 infection after the occurrence of reverse transcription but before integration [8,21,22]. This evidence suggested that MxB might be interfering with one or more of the following processes: 1) HIV-1 uncoating, 2) nuclear import of the HIV-1 pre-integration complex, or 3) nuclear maturation of the pre-integration complex. However, the mechanism by which MxB interferes with early steps of HIV-1 infection is not understood.

This work explores the ability of MxB to bind to the HIV-1 core in vitro and during infection. We showed that MxB interacts with in vitro assembled HIV-1 capsid-nucleocapsid (CA-NC) complexes, which recapitulate the surface of the HIV-1 core. In agreement, we found that MxB associates with HIV-1 cores during infection using the fate of the capsid assay. Remarkably, the binding of MxB to the HIV-1 core inhibits the uncoating process of HIV-1 defining MxB as an endogenously expressed protein that prevents HIV-1 uncoating. To find small-molecule inhibitors that prevent the binding of MxB to the HIV-1 core, we screened a battery of structurally well-known HIV-1 capsid inhibitors for their ability to prevent the binding of MxB to the HIV-1 core. Interestingly, a benzimidazole-based compound known to have a binding pocket on the surface of the HIV-1 capsid prevents the binding of MxB to the capsid. These experiments suggested an overlap between the capsid binding sites for MxB and the benzimidazole-based compound. Assaying the contribution of the different MxB protein domains to capsid binding and restriction revealed that the 20 N-terminal amino acids are responsible for the ability of MxB to bind to the HIV-1 core. In addition, we provide evidence that the C-terminal leucine zipper domain of MxB provides the necessary avidity for the interaction of MxB with the HIV-1 core. Overall, our studies showed that MxB binds to the HIV-1 core and inhibits the uncoating process of HIV-1 leading to an infection block.

## Results

### MxB binds in vitro assembled HIV-1 CA-NC complexes

**Figure 1:**
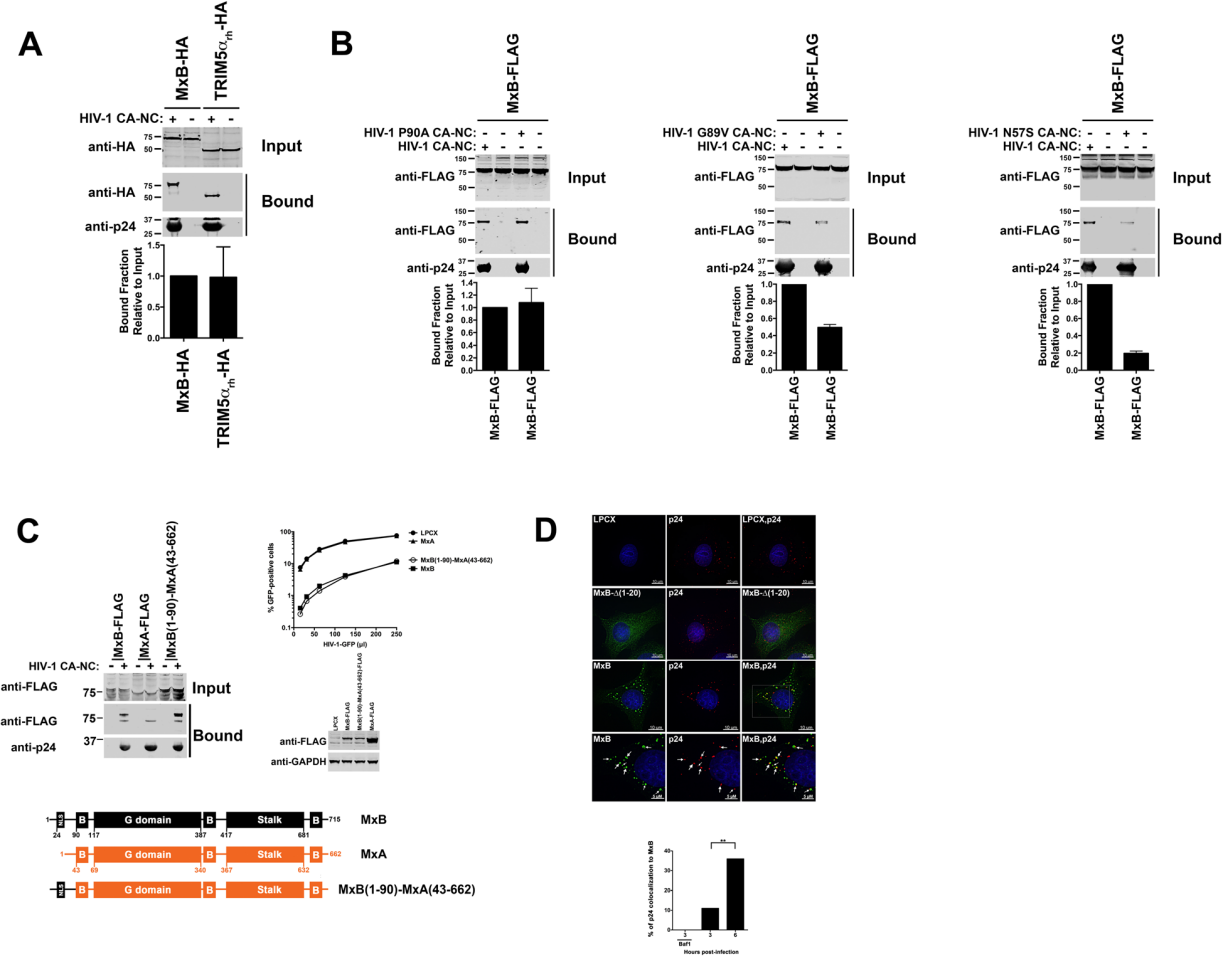
**MxB binds in vitro assembled HIV-1 CA-NC complexes. (A)** The ability of MxB to bind in vitro assembled HIV-1 CA-NC complexes was measured as described in Methods. **Input** and **Bound** fractions were analyzed by Western blotting using anti-HA or anti-p24 antibodies. Similar results were obtained in three independent experiments, and the standard deviation for the bound fraction relative to input is shown. **(B)** The ability of MxB to bind in vitro assembled HIV-1 CA-NC complexes’ bearing the capsid changes P90A, G89V and N57S was measured as described above. Similar results were obtained in three independent experiments. **(C)** The ability of MxA, MxB and the protein chimera MxB (1–90)-MxA (43–662) to bind in vitro assembled HIV-1 CA-NC were measured as described above (left panel). Cf2Th cells stably expressing MxA, MxB, or MxB (1–90)-MxA (43–662) were challenged with increasing amounts of HIV-1-GFP viruses. Infection was determined by measuring the percentage of GFP-positive cells (right panel). MxA, MxB and MxB (1–90)-MxA (43–662) proteins are depicted (lower panel). Similar results were obtained in three independent experiments. NLS: nuclear localization signal. B: comprises the bundle signaling element. G: contains a GTPase domain. Stalk: domain that mediates assembly into oligomers. **(D)** Increased HIV-1 capsid colocalization with MxB upon infection. CF2Th cells stably expressing MxB, MxB-∆(1–20), or containing the empty vector LPCX, were spinoculated with VSV-G pseudotyped R7ΔEnv virions for 2 h. Cells were fixed 3 and 6 hours post-infection. Colocalization of MxB with HIV capsid (p24) is shown 6 hours post infection. The percentage of p24 associated with MxB was quantified by counting the number of p24 positive virions, which are positive for MxB is shown. Between 20–25 images were analyzed per condition using the Imaris Software as described in Methods. Bafilomycin A1 (Baf1). Data is representative of 3 independent experiments. **, P <0.05.

To test the ability of MxB to restrict HIV-1 infection, we stably expressed FLAG-tagged MxB in human HeLa, human U937 and dog Cf2Th cells using the LPCX vector system and tested for the ability of these cells to restrict HIV-1 and other retroviruses (Additional file 1A, B and C), as described [23]. In agreement with recent publications [8,20-22], the long form of MxB potently blocks HIV-1 infection in all tested cell lines. Because the use of HIV-1 viruses bearing capsid changes suggested that the HIV-1 capsid is the determinant for MxB restriction, we examined the ability of MxB to associate with the HIV-1 core. For this purpose, we tested the ability of MxB to bind in vitro assembled HIV-1 capsid-nucleocapsid (CA-NC) complexes, as described [24-26]. In vitro assembled HIV-1 CA-NC complexes recapitulate the surface of the HIV-1 core, and are an established model to evaluate the ability of cellular factors to interact with the HIV-1 core [25,27-29]. As shown in Figure 1A, MxB binds to in vitro assembled HIV-1 CA-NC complexes in a similar manner when compared to the HIV-1 capsid interacting molecule rhesus TRIM5α (TRIM5α_rh_) [25,29]. Stable expression of MxB or TRIM5α_rh_ potently blocked HIV-1 infection (Additional file 2A).

To further test the role of MxB binding to HIV-1 capsid in restriction, we tested the ability of MxB to bind in vitro assembled HIV-1 CA-NC complexes bearing the capsid changes P90A, G89V or N57S, which when incorporated into the virus allow HIV-1 to partially escape MxB restriction [8,21,22]. As shown in Figure 1B, MxB binds to in vitro assembled HIV-1 CA-NC complexes bearing the capsid change P90A, suggesting that binding is necessary but not sufficient for restriction. Interestingly, MxB bound less to in vitro assembled HIV-1 CA-NC complexes bearing changes G89V and N57S when compared to wild type complexes (Figure 1B). As previously shown, HIV-1 bearing the capsid change P90A, G89V or N57S partially escaped MxB restriction (Additional file 2B). Notwithstanding HIV-1 viruses bearing the capsid change P90A, G89V or N57S partially escape MxB restriction, we found that P90A is the only capsid change that did not result in a virus with an infectivity defect (Additional file 2C). To confirm the bona fide origin and functionality of in vitro assembled HIV-1 CA-NC complexes tubes bearing capsid mutants P90A, G89V and N57S, we tested the binding of cleavage and polyadenylation specific factor 6 (CPSF6) to the different capsid mutants. CPSF6 was able to bind in vitro assembled HIV-1 CA-NC complexes bearing changes P90A and G89V, but not N57S (Additional file 2D). In addition, we showed the ability of TRIM5α_rh_ to bind in vitro assembled HIV-1 CA-NC complexes bearing the change N57S (Additional file 2D).

Furthermore, we performed electron microscopy to show that the in vitro assembled HIV-1 CA-NC mutants form similar tubular structures when compared to wild type (Additional file 2E).

Next we tested whether MxA, which is the closest human homolog to MxB that contains antiviral activity [2], binds to in vitro assembled HIV-1 CA-NC complexes. MxB is composed of the same domains when compared to MxA with the exception that MxB exhibits a longer N-terminal domain (Figure 1C, bottom panel) [5]. Interestingly, MxA was not able to interact with in vitro assembled HIV-1 CA-NC complexes when compared to MxB (Figure 1C, upper left panel). In agreement, MxA was not able to block HIV-1 infection when compared to MxB (Figure 1C, upper right panel) [8]. Next we tested the capsid binding ability of a protein chimera containing residues 1–90 of MxB fused to residues 43–662 of MxA [MxB (1–90)-MxA (43–662)]. Contrary to MxA, the protein chimera MxB (1–90)-MxA (43–662) gained the ability to bind in vitro assembled HIV-1 CA-NC complexes (Figure 1C, left panel). These results suggested that the N-terminal region of MxB is involved in its ability to bind capsid. In addition, MxB (1–90)-MxA (43–662) gained the ability to block HIV-1 infection (Figure 1C, right panel). Furthermore, these results correlated the ability of MxB to bind capsid with HIV-1 restriction.

Next we measured the ability of HIV-1 cores to colocalize with MxB in living cells using fluorescence microscopy over time, as described [30]. For this purpose, we infected Cf2Th cells stably expressing MxB with VSV-G pseudotyped R7∆Env HIV-1 viruses in the presence or absence of Bafilomycin A1, which inhibits the entry of pH-dependent viruses [31]. At 6 hours post-infection, samples were fixed and MxB colocalization with HIV-1 cores was quantified (Figure 1D). The percentage of p24 that colocalizes with MxB was calculated as the number of p24 positive virions that are associated with MxB as described in Methods (Figure 1D, lower panel). As a control, we performed similar experiments in cells expressing MxB-∆(1–20), which is a deletion mutant that no longer binds capsid or restrict HIV-1 (see below). Colocalization between MxB-∆(1–20) and p24 was not observed. These results showed an increase in p24 colocalization with MxB over time suggesting that MxB directly associates with HIV-1 cores in living cells.

In agreement with the fact that MxB modestly blocks SIVmac and HIV-2 infection (Additional file 1C), we found that MxB binds to in vitro assembled SIVmac and HIV-2 CA-NC complexes (Additional file 1D).

Overall, these results showed that MxB binds to HIV-1 capsid, and that binding to capsid is necessary for the ability of MxB to block HIV-1 infection. By the use of the protein chimera MxB (1–90)-MxA (43–662), we have correlated binding with restriction. Additionally, we showed that the N-terminal 90 amino acids of MxB are important for the ability of MxB to bind the HIV-1 capsid and restriction of HIV-1 infection.

### MxB binds to the HIV-1 core and inhibits the uncoating process during infection

**Figure 2:**
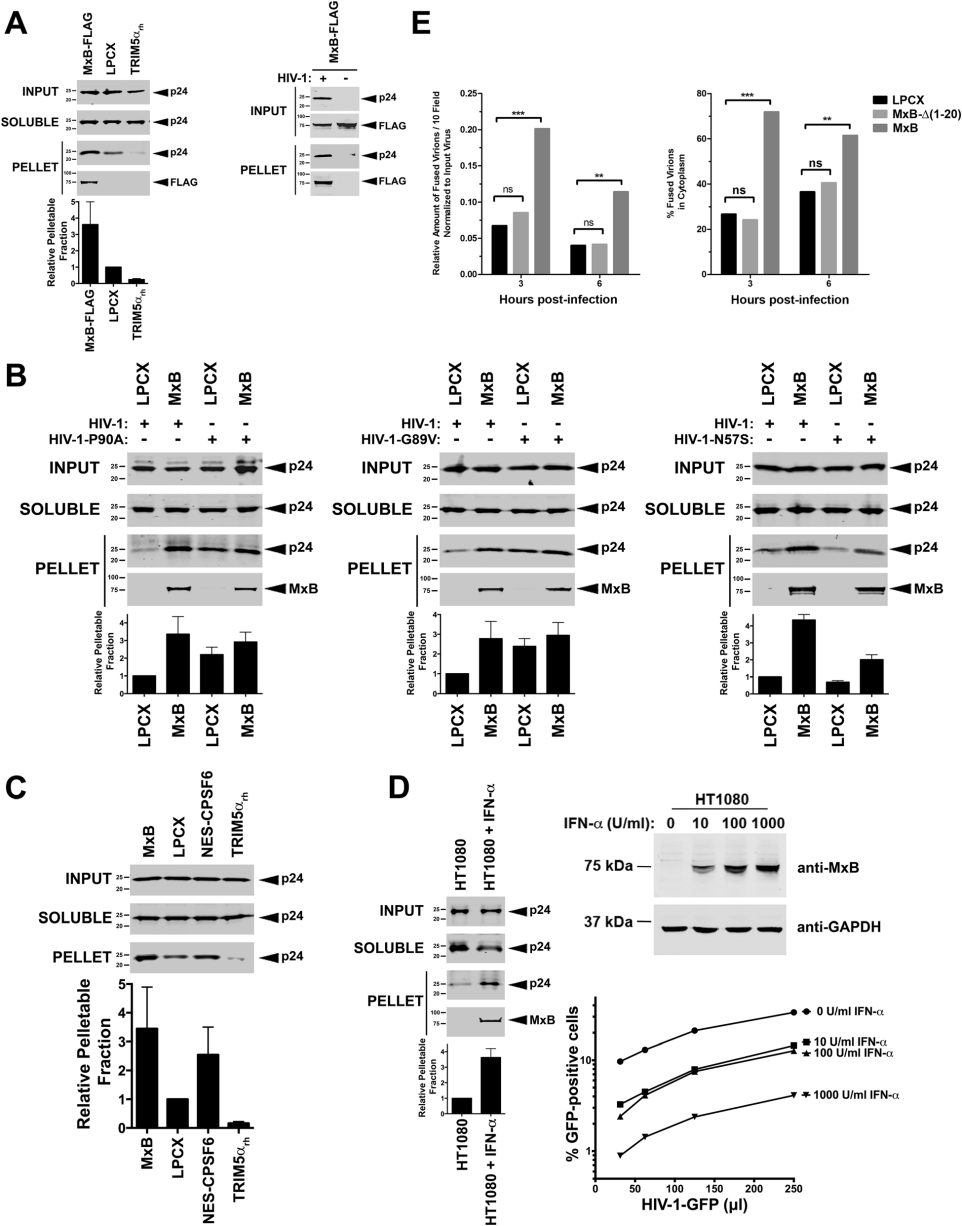
**MxB binds to the HIV-1 core and inhibits the uncoating process of HIV-1 during infection. (A)** Cf2Th cells stably expressing FLAG-tagged MxB (MxB-FLAG), TRIM5α_rh_ or containing the empty vector LPCX were challenged using similar amounts of HIV-1. Sixteen hours post-infection samples were processed for the fate of the capsid assay as described in Methods. Input, soluble and pellet fractions were analyzed by Western blotting using anti-p24 antibodies (left panel). The binding of MxB to the viral core was assayed by Western blotting using anti-FLAG antibodies (left panel). The quantification of pelletable fractions relative to the pellet found when infecting cells containing the LPCX control vector by wild type HIV-1 was calculated for each experiment. Similar results were obtained in three independent experiments, and the standard deviations are shown. To control for the presence of MxB-FLAG in pelletable fractions when using mock-infected cells, Cf2Th cells stably expressing MxB-FLAG were challenged with HIV-1 or not, and processed for the fate of the capsid assay (right panel). Input and pellet fractions were analyzed by Western blotting using anti-p24 and anti-FLAG antibodies. Similar results were obtained in three independent experiments and a representative experiment is shown. **(B)** Cf2Th cells stably expressing MxB or stably transduced with the empty vector LPCX were challenged using similar amounts of HIV-1-P90A (left panel), HIV-1-G89V (middle panel) or HIV-1-N57S (right panel). Sixteen hours post-infection samples were processed for the fate of the capsid assay. Input, soluble and pellet fractions were analyzed by Western blotting using anti-p24 antibodies. The quantification of pelletable fractions relative to the pellet found when infecting cells containing the LPCX control vector by wild type HIV-1 was calculated for each experiment. Similar results were obtained in three independent experiments, and the standard deviations are shown. **(C)** Cf2Th cells stably expressing MxB, NES-CPSF6, TRIM5α_rh_ or containing the empty vector LPCX were challenged using similar amounts of HIV-1. Sixteen hours post-infection samples were processed for the fate of the capsid assay. Input, soluble and pellet fractions were analyzed by Western blotting using anti-p24 antibodies. Similar results were obtained in three independent experiments, and the standard deviation for pelletable fractions relative to the pellet found in cells containing the empty vector LPCX are shown. **(D)** IFN-α-treated (1000U/ml for 24 hours) or untreated Human HT1080 cells were challenged using similar amounts of HIV-1. Sixteen hours post-infection samples were processed for the fate of the capsid assay (left panel). Quantification of pelletable fractions relative to the pellet found when infecting untreated HT1080 cells by wild type HIV-1 was calculated for each experiment. Similar results were obtained in three independent experiments, and the standard deviations are shown. Induction of MxB expression in human HT1080 cells by the indicated amounts of IFN-α after 24 hours of exposure is shown (upper right panel). The ability of IFN-α-treated HT1080 cells to block HIV-1 infection is shown (bottom right panel). **(E)** MxB causes retention of HIV virions in the cytoplasm. Cf2Th cells stably expressing MxB, MxB-∆(1–20), or containing the empty vector LPCX, were spinoculated with fluorescently labeled HIV-1 virions for 2 h. HIV-1 viruses were fluorescently labeled with Vpr-GFP (label the inside of the virus) and the S15-mCherry (labels the lipid membrane of the virus). Cells were fixed and analyzed 3 and 6 hours post-infection. The total number of fused viruses (S15-mCherry negative particles) is shown, as determined by counting 10 fields using the Imaris software. On the left panel, the relative number of fused virions normalized to input viruses overtime are shown. On the right panel, the percentage of total fused virions retained in the cytoplasm overtime is shown. Results are representative of 3 independent experiments. ***, P <0.001 and **, P <0.05, as compared with respective controls.

To investigate whether MxB interacts with the HIV-1 core during infection, we performed the fate of the capsid assay to follow the protein composition of HIV-1 cores during infection. The fate of the capsid assay examines the uncoating process of HIV-1 and measures the amount of soluble versus pelletable capsids (HIV-1 cores) during infection in living cells, as described [25,28,32]. Input, soluble and pellet fractions were analyzed by Western blotting using antibodies against p24. Pellet fractions were also analyzed for the presence of MxB by Western blotting using anti-FLAG antibodies. As shown in Figure 2A (left panel), MxB is present on HIV-1 cores isolated from HIV-1 infected Cf2Th cells stably expressing MxB. As a control, we observed the absence of MxB in pelletable fractions prepared from uninfected control cells (Figure 2A, right panel). These results suggested that MxB interacts with the HIV-1 core during infection. Remarkably, we noticed an increase in the amount of pelletable capsid when comparing cells stably expressing MxB with cells containing the empty vector LPCX (Figure 2A, left panel). These experiments suggested that MxB inhibits the uncoating process of HIV-1. In parallel, we performed the fate of the capsid in cells stably expressing TRIM5α_rh_, which accelerates uncoating (Figure 2A) [25,32].

Consequently, we tested the ability of MxB to inhibit the uncoating process of HIV-1 mutants that partially overcome restriction. For this purpose, we challenged Cf2Th cells stably expressing MxB or containing the empty vector LPCX with similar amounts of HIV-1-P90A, HIV-1-G89V or HIV-1-N57S viruses (Figure 2B), which partially escape MxB restriction [8,21,22]. MxB only minimally inhibits the uncoating process of HIV-1 viruses bearing capsid changes P90A, G89V and N57S (Figure 2B). Accordingly, MxB partially affected the infection of HIV-1-P90A, HIV-1-G89V and HIV-1-N57S viruses. In agreement with our binding assays, we also observed that HIV-1 cores bearing the different capsid mutations were able to recruit MxB.

We and others have previously observed that cells expressing the CPSF6 protein fused to a nuclear export signal (NES-CPSF6) inhibits the uncoating process of HIV-1 [33,34]; therefore, we compared the abilities of MxB and NES-CPSF6 to inhibit the uncoating process of HIV-1. As shown in Figure 2C, MxB is as potent as NES-CPSF6 in inhibiting the uncoating process of HIV-1. As expected, MxB, NES-CPSF6 and TRIM5α_rh_ potently restricted HIV-1 infection (Additional file 2F).

Next we tested the ability of IFN-α-treated human HT1080 cells to inhibit the uncoating process of HIV-1. For this purpose, we challenged human HT1080 cells that were treated or not with IFN-α with similar amounts of HIV-1-GFP and performed the fate of the capsid assay. As shown in Figure 2D, IFN-α-treated HT1080 cells inhibit the uncoating process of HIV-1. These findings suggested that IFN-α-treated HT1080 cells prevent the uncoating process of HIV-1, which could be mediated by any of the IFN-α inducible genes. In agreement with our previous findings, endogenously expressed MxB co-sedimented with pelletable capsids suggesting that endogenously expressed MxB interacts with the HIV-1 core in living cells. As expected, IFN-α-treated human HT1080 expressed endogenous MxB and potently blocked HIV-1 infection (Figure 2D, right panels).

Subsequently, we tested whether MxB is able to retain HIV-1 cores in the cytoplasm of infected living cells by fluorescence microscopy. For this purpose, we challenged Cf2Th cells stably expressing MxB, MxB-∆(1–20), or containing the empty vector LPCX, with dually labeled HIV-1 particles containing Vpr-GFP and the S15-mCherry, as described [30]. Infected cells were fixed at the indicated time points, and the number of fused virions (S15-mCherry negative particles) was counted in 10 different visual fields. As shown on Figure 2E, infected Cf2Th cells expressing MxB allowed the accumulation of viral cores over time when compared to infected Cf2Th stably cells expressing MxB-∆(1–20) or containing the empty vector LPCX. These results suggested that expression of MxB allows the accumulation of HIV-1 cores in the cytoplasm over time.

### The interaction between MxB and the HIV-1 core is prevented by a benzimidazole-based inhibitor that binds to a specific pocket in the HIV-1 capsid

**Figure 3:**
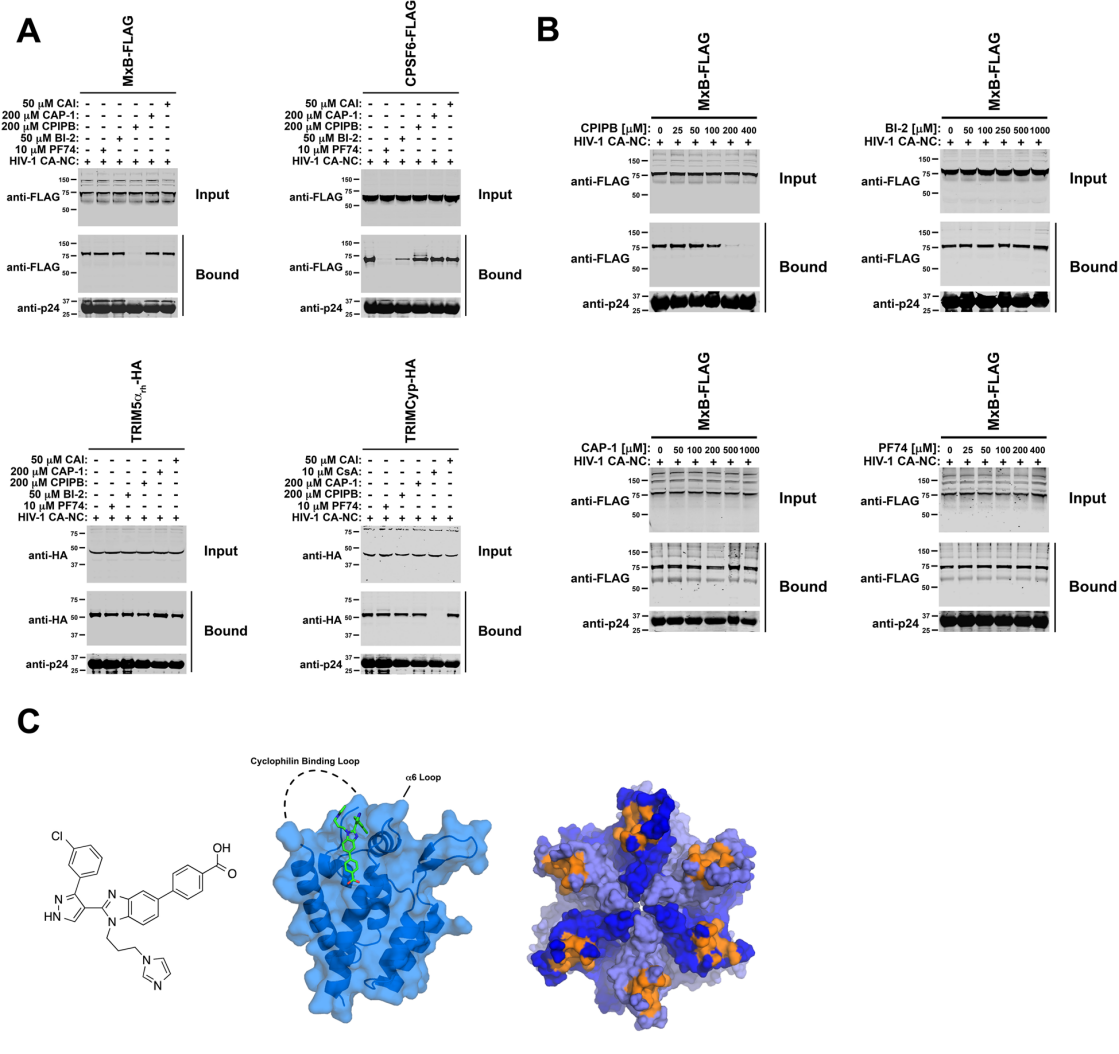
**The interaction of MxB with the HIV-core is inhibited by a small-molecule that binds into a pocket between the bases of the Cyp A binding loop and the loop that connects helices 6 and 7 of HIV-1 capsid. (A)** The ability of MxB-FLAG (upper left panel), CPSF6-FLAG (upper right panel), TRIM5α_rh_-HA (lower left panel), and TRIMCyp-HA (lower right panel) to bind in vitro assembled HIV-1 CA-NC complexes was measured in the presence of the indicated concentration of the small molecules PF74, BI-2, CPIPB (4-{2-[3-(3-clorophenyl)-1H-pyrazol-4-yl]-1-[3-(1H-imidazol-1-yl-propyl]-1H-benzimidazol-5-yl} benzoic acid), CAP-1 and the peptide CAI, as described in Methods. Similar results were obtained in three independent experiments and a representative experiment is shown. **(B)** The ability of MxB to bind in vitro assembled HIV-1 CA-NC complexes in the presence of the indicated concentrations of the small molecule CPIPB (upper left panel), BI-2 (upper right panel), CAP-1 (bottom left panel) and PF74 (bottom right panel) was measured. Similar results were obtained in three independent experiments and a representative experiment is shown. **(C)** Chemical structure of the small molecule CPIPB is shown (left panel). Surface representation of the X-ray structure of the HIV-1 capsid in complex with CPIPB (PDB entry 4E91) (middle panel). Surface representation of the X-ray structure of the HIV-1 CA hexamer (PDB entry 3H4E) showing in orange residues that directly interact with CPIPB on the surface of the HIV-1 capsid.

To better understand the binding of MxB to the HIV-1 core, we tested the ability of a battery of small molecules that target capsid to prevent the binding of MxB to in vitro assembled HIV-1 CA-NC complexes [35]. For this purpose, we examined the binding of MxB to in vitro assembled HIV-1 CA-NC complexes in the presence of small-molecules that target HIV-1 capsid. In these experiments, we used PF74 [35-37], BI-2 [35,38], CAP-1 [35,39,40], 4-{2-[3-(3-chlorophenyl)-1H-pyrazol-4-yl]-1-[3-(1H-imidazol-1-yl) propyl]-1H-benzimidazol-5-yl} benzoic acid dihydrochloride (CPIPB) [41,42], and the peptide CAI [35,43]. Interestingly, only CPIPB inhibited the ability of MxB to bind in vitro assembled HIV-1 CA-NC complexes (Figure 3A, upper left panel). As a control, we tested the ability of these compounds to block the binding of CPSF6, TRIM5α_rh_ and TRIMCyp to in vitro assembled HIV-1 CA-NC complexes (Figure 3A). As expected, the drugs PF74 and BI-2 affected the ability of CPSF6 to bind capsid due to the fact that PF74, BI-2 and CPSF6 bind to similar regions on the HIV-1 capsid (Figure 3A) [33,44]. Similarly, the binding of TRIMCyp to capsid was only affected by cyclosporine A (CsA), which is an inhibitor of cyclophilin A (Cyp A) (Figure 3A). To further understand the ability of CPIPB to prevent the binding of MxB to in vitro assembled HIV-1 CA-NC complexes, we performed a dose–response curve for the different drugs (Figure 3B). We observed that using CPIPB at 200 μM completely inhibited the binding of MxB to capsid. As a control, we showed that the use of BI-2, CAP-1 or PF74 at 200 μM or higher concentrations did not affect the binding of MxB to capsid. These experiments suggested that the capsid-binding site for MxB overlaps with the binding pocket for CPIPB, which is located between the base of the Cyp A binding loop and the loop that connects helices 6 and 7 of HIV-1 capsid (Figure 3C). The fact that the HIV-1 capsid binding site for MxB is next to the base of the Cyp A binding loop is in agreement with previous observations suggesting that CsA partially relieves MxB restriction to HIV-1 and that HIV-1-P90A and HIV-1-G89V viruses partially escape restriction [8,22]. Although CsA partially relieves MxB restriction (Figure), it did not affect the ability of MxB to bind in vitro assembled HIV-1 CA-NC complexes (Additional file 3B). As a control, we observed that CsA relieved TRIMCyp restriction and binding to capsid (Additional file 3A and B). In addition, we tested whether MxB recruits Cyp A to the HIV-1 capsid. As shown in Additional file 3C, MxB did not increase the amount of endogenous Cyp A recruited to the capsid. As a control, we showed that the use CsA prevented the binding of endogenous Cyp A to the HIV-1 capsid (Additional file 3C). Altogether these results suggested that the binding of MxB to HIV-1 capsid is specific and can be modulated by a small-molecule inhibitor.

Next we tested the ability of CPIPB to release HIV-1 restriction in mammalian cells expressing MxB. However, we could not measure release of restriction since we found that CPIPB is highly toxic for mammalian cells above 50 μM.

### Contribution of the different protein domains of MxB to HIV-1 capsid-binding and restriction

**Figure 4:**
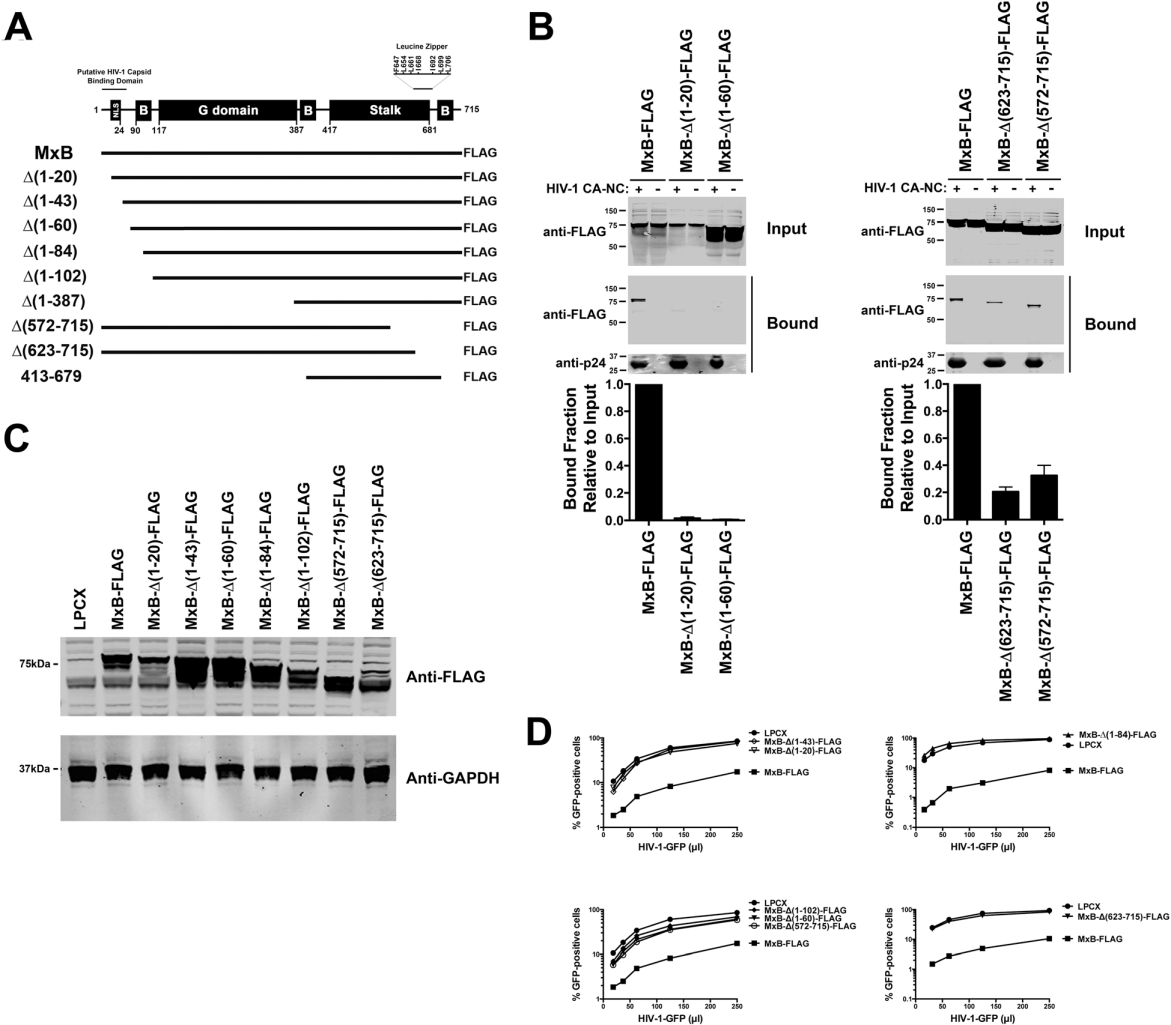
**Contribution of the different domains of MxB to capsid binding and restriction of HIV-1. (A)** Wild type human MxB protein is depicted in the top figure. The numbers of the amino acid residues at the boundaries of the MxB domains are indicated. The different MxB variants are shown. **(B)** The ability of wild type and mutant MxB proteins to bind in vitro assembled HIV-1 CA-NC complexes was measured, as described in Methods. Similar results were obtained in three independent experiments, and the standard deviation for bound fractions relative to input are shown. **(C)** Cf2Th cells stably expressing wild type and the indicated MxB variant were analyzed by Western blotting using anti-FLAG antibodies. As loading control, cell lysates were Western blotted using anti-GAPDH antibodies. **(D)** Cf2Th cells stably expressing wild type and the indicated MxB variants were challenged with increasing amounts of HIV-1 viruses expressing GFP as a reporter for infection (HIV-1-GFP). Forty-eight hours post-infection the percentage of GFP-positive cells was determined by flow cytometry. As a control, Cf2Th cells stably transduced with the empty vector LPCX were challenged with increasing amounts of HIV-1-GFP. Similar results were obtained in three independent experiments and a representative experiment is shown.

MxB contains several different protein regions: a nuclear localization signal (NLS), G domain, stalk region, and a leucine zipper motif (Figure 4A). To understand the contribution of the different MxB domains to capsid-binding and restriction, we generated a series of deletion constructs (Figure 4A and Table 1). Our observations revealed that deletion of the 20 N-terminal amino acids of MxB abolished the ability of the protein to bind in vitro assembled HIV-1 CA-NC complexes (Figure 4B, Additional file 4 and Table 1). These results are in agreement with the ability of MxB (1–90)-MxA (43–662) to bind capsid, which suggests that the N-terminal residues of MxB are involved in its ability to bind HIV-1 capsid. As shown in Figure 4B, deletion of the C-terminal domain of MxB, which contains a leucine zipper, decreased the ability of MxB to bind in vitro assembled HIV-1 CA-NC complexes. Next we tested the ability of the different MxB variants to block HIV-1 infection. For this purpose, we stably expressed the different MxB variants in Cf2Th cells (Figure 4C), and challenged them using increasing amounts of HIV-1-GFP (Figure 4D). Interestingly, N-terminal or C-terminal deletions affected the ability of MxB to block HIV-1 infection. These results suggested that the N-terminal and C-terminal domain of MxB are important to block HIV-1 infection.

**Table 1:** **Phenotypes of MxB variants**

**MxB variant**	**Restriction of HIV-1** ^**a**^	**Binding to HIV-1**	**Oligomerization** ^**c**^
		**CA-NC Complexes** ^**b**^	
WT	+	+++	+
Δ (1–20)	-	-	+
Δ (1–43)	-	-	ND
Δ (1–60)	-	-	+
Δ (1–84)	-	-	ND
Δ (1–102)	-	ND	ND
Δ (1–387)	ND	-	ND
Δ (572–715)	-	+	-
Δ (623–715)	-	+	-
413-679	ND	-	ND
MxB (1–90)-MxA (43–662)	+	+++	ND
L661k	-	+	-

WT, Wild type.

ND, Not determined.

^a^Restriction was measured by infecting cells expressing the indicated MxB variant with HIV-1-GFP. After 48 hours, the percentage of GFP-positive cells (infected cells) was determined by flow cytometry. “+”: indicates restriction, “-”: indicates absence of restriction. ND: Not Determined.

^b^Binding to the HIV-1 capsid complexes was determined for each MxB variant as described in Methods. “+++”: indicates 100% binding which corresponds to the amount of wild type MxB bound to HIV-1 CA-NC complexes, “+”: indicates ~10% binding compared to wild type MxB when expressed at similar levels, “-”: indicates no binding. Experiments were repeated at least three times, and standard deviations are shown in Figure legends.

^c^Wild type and mutant MxB-FLAG proteins were assayed for association with wild-type MxB-HA as described in Methods. “+”: oligomerization observed in wild-type MxB when expressed at similar levels. ND: Not Determined.

### Contribution of oligomerization to the ability of MxB to bind to the HIV-1 core

**Figure 5:**
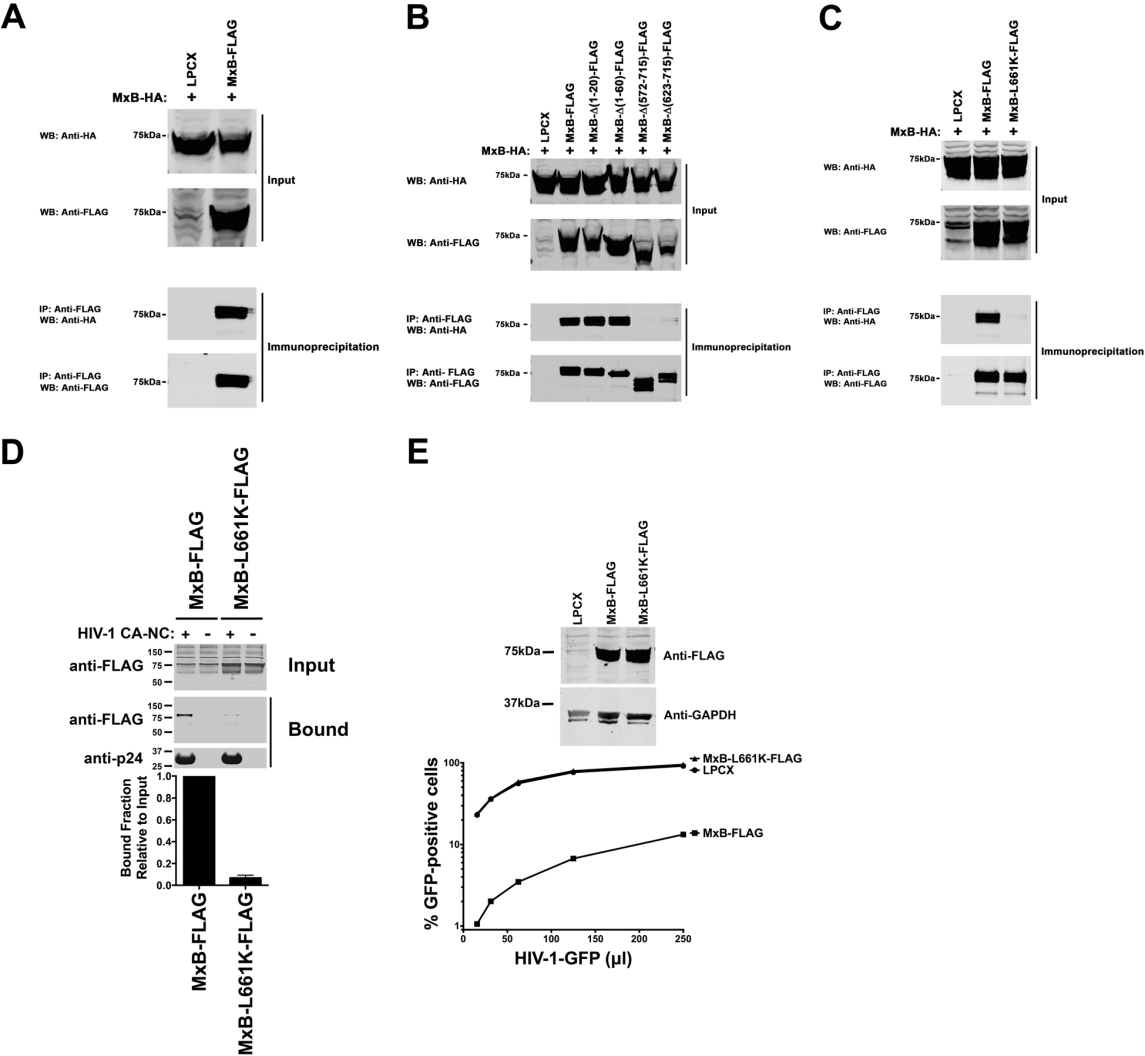
**Contribution of oligomerization to the binding of MxB to the HIV-1 core. (A)** MxB oligomerization assay. Human 293T cells were cotransfected with plasmids expressing wild type MxB-HA and MxB-FLAG proteins. Cells were lysed 24 hours post-transfection and analyzed by Western blotting using anti-HA and anti-FLAG antibodies (Input). Subsequently, lysates were immunoprecipitated by using anti-FLAG agarose beads, as described in Methods. Anti-FLAG agarose beads were eluted using the 3X FLAG peptide. Elutions were analyzed by Western blotting using anti-HA and anti-FLAG antibodies (Immunoprecipitation). WB, Western blot; IP, Immunoprecipitation; WT, wild type. **(B)** Oligomerization of MxB variants. Human 293T cells were cotransfected with a plasmid expressing wild type MxB-HA and a plasmid either expressing wild type or the indicated mutant MxB-FLAG protein. Similarly, FLAG-tagged proteins were immunoprecipitated, and elutions were analyzed by Western blotting using anti-HA and anti-FLAG antibodies. **(C)** Oligomerization of the MxB’s leucine zipper domain mutant L661K. Human 293T cells were cotransfected with a plasmid expressing wild type MxB-HA and a plasmid either expressing wild type or the mutant MxB-L661K-FLAG protein. Similarly, FLAG-tagged proteins were immunoprecipitated, and elutions were analyzed by Western blotting using anti-HA and anti-FLAG antibodies. **(D)** The ability of MxB-L661K-FLAG to bind in vitro assembled HIV-1 CA-NC complexes was measured, as described Methods. **(E)** Cf2Th cells stably expressing wild type or the indicated mutant MxB protein (upper panel) were challenged with increasing amounts of HIV-1-GFP. Forty-eight hours post-infection the percentage of GFP-positive cells was measured by flow cytometry (lower panel). As a control, Cf2Th cells stably transduced with the empty vector LPCX were challenged with HIV-1. Similar results were obtained in three independent experiments and a representative experiment is shown.

To examine the contribution of oligomerization to the ability of MxB to bind capsid and restrict HIV-1, we tested the oligomerization ability of the different MxB variants. We evaluated the biochemical ability of MxB containing a FLAG tag (MxB-FLAG) to interact with MxB containing an HA tag (MxB-HA). For this purpose, we cotransfected cells using similar amounts of both MxB-FLAG and MxB-HA constructs. Cells were lysed, and MxB-FLAG was immunoprecipitated by using anti-FLAG beads, as described in Methods. Proteins eluted by the FLAG peptide were analyzed by Western blotting using anti-HA and anti-FLAG antibodies (Figure 5A). In agreement with the notion that MxB forms oligomers, MxB-FLAG interacts with MxB-HA (Figure 5A). These results showed that MxB forms oligomers when expressed in mammalian cells, as it has been shown using the same methodology for MxA [11]. Next we measured the ability of the different MxB-FLAG variants to oligomerize with MxB-HA (Figure 5B). After testing all the variants that exhibited comparable expression to wild type MxB, we found that only MxB variants ∆(572–715) and ∆(623–715) lost their ability to oligomerize (Figure 5B and Table 1), suggesting that the C-terminal residues of MxB are involved in oligomerization. These results are in agreement with the presence of a leucine zipper motif in the C-terminal region of MxB (Figure 4A); this leucine zipper has been postulated to be important for the oligomerization ability of MxA [45,46]. Therefore, we disrupted the leucine zipper of MxB by a changing a single amino (L661K). Disruption of the leucine zipper by a single point mutation disrupted the ability of MxB to oligomerize (Figure 5C). In agreement with our deletion experiments, MxB-L661K neither bound in vitro assembled HIV-1 CA-NC complexes nor restricted HIV-1 infection (Figure 5D and E). These results suggested that oligomerization provides the necessary avidity for MxB to bind HIV-1 capsid and is required for restriction (Table 1).

## Discussion

The present work explores the ability of MxB to bind to the HIV-1 core, and the contribution of binding to HIV-1 restriction. Our findings provided novel insights into the mechanism used by MxB to block HIV-1 infection: 1) MxB binds to the HIV-1 capsid, 2) MxB colocalizes with HIV-1 cores in infected cells as determined by fluorescence microscopy, 3) MxB is associated biochemically with pelletable cores during infection, 4) MxB inhibits the uncoating process of HIV-1 during infection as measured by the fate of the capsid assay and a microscopy-based assay, 5) the small-molecule inhibitor CPIPB, which binds into a pocket between the bases of the Cyp A binding loop and the loop that connects helices 6 and 7 of HIV-1 capsid (Figure 3C) [41,42], interfered with the ability of MxB to bind HIV-1 capsid, 6) domain mapping experiments and the use of a protein chimera showed that capsid binding correlates with restriction, and 6) disruption of MxB’s leucine zipper motif abrogated the ability of MxB to oligomerize, to bind capsid and to block HIV-1 infection.

We tested the ability of MxB to bind in vitro assembled HIV-1 CA-NC complexes, and showed that MxB binds to the HIV-1 capsid in a similar manner when compared to the restriction factor TRIM5α_rh_ [25,29]. The testing of MxB binding to capsid mutants P90A, G89V and N57S revealed that binding is necessary but not sufficient for restriction. By using the fate of the capsid assay, we also showed that MxB is contained in pelletable fractions suggesting that MxB associates with the HIV-1 core during infection. In agreement, fluorescence microscopy experiments showed that colocalization of HIV-1 cores with MxB increased over time in infected cells suggesting that MxB associates with the HIV-1 core.

We also compared the capsid-binding abilities of MxB with MxA, which does not restrict HIV-1 infection [6,8]. Our experiments showed that MxA does not bind in vitro assembled HIV-1 CA-NC complexes. MxA and MxB exhibit similar sequences and domains suggesting analogous functions [1,11-14]. Interestingly, the difference between MxA and MxB is that the latter contains an additional 60 amino acids on the N-terminal end, which might be directly involved in the binding of MxB to capsid. In agreement with this hypothesis, we showed that the protein chimera MxB (1–90)-MxA (43–662) gains the ability to bind HIV-1 capsid and to restrict HIV-1 when compared to MxA. These results suggested that the N-terminal residues of MxB are involved in the ability of MxB to bind capsid and restrict HIV-1.

This work shows that MxB inhibits the HIV-1 uncoating process during infection. Using the fate of the capsid assay, which analyses the HIV-1 uncoating process during infection [25,28,47], we showed that MxB stabilizes the HIV-1 core during infection. In agreement with these observations, we also showed that MxB facilitates the accumulation of HIV-1 cores over time by fluorescence microscopy in living cells. Overall our results showed that the IFN-α inducible MxB protein is a naturally expressed protein that inhibits the uncoating process of HIV-1.

To explore MxB’s binding site on the surface of the HIV-1 capsid, we tested a battery of capsid inhibitors that are well studied with respect to their binding sites on the HIV-1 capsid protein. From these investigations, we found that the compound CPIPB was able to compete with MxB for binding to capsid suggesting that the binding pocket of CPIPB overlaps with the binding site for MxB [41,42]. The binding pocket for CPIPB is located between the base of the Cyp A binding loop and the loop that connects helices 6 and 7 of HIV-1 capsid (Figure 3C). Remarkably, CPIPB binding to capsid facilitates crystallization of capsid by decreasing the mobility of the Cyp A binding loop and the loop that connects helices 6 and 7 of HIV-1 capsid [41,42]. In agreement, the binding of MxB to the HIV-1 capsid stabilizes the HIV-1 core suggesting that this particular capsid region modulates stability of the HIV-1 core.

Domain mapping studies showed that the 20 N-terminal amino acids are essential for the ability of MxB to bind HIV-1 capsid and restrict infection. Although MxB-∆(1–20) did not bind capsid or restrict HIV-1, it retained its oligomerization ability. These experiments suggested that the N-terminal domain is important for the ability of MxB to restrict HIV-1 infection. Future experiments destined to understand the structure of this region will shed light on the domain used by MxB to interact with the HIV-1 core.

Finally, we established that MxB’s oligomerization by its leucine zipper domain is important for its ability to bind capsid and restrict infection. Deletion and point mutations of MxB’s leucine zipper motif suggested that oligomerization contributes to MxB’s binding avidity for the HIV-1 core.

**Figure 6:**
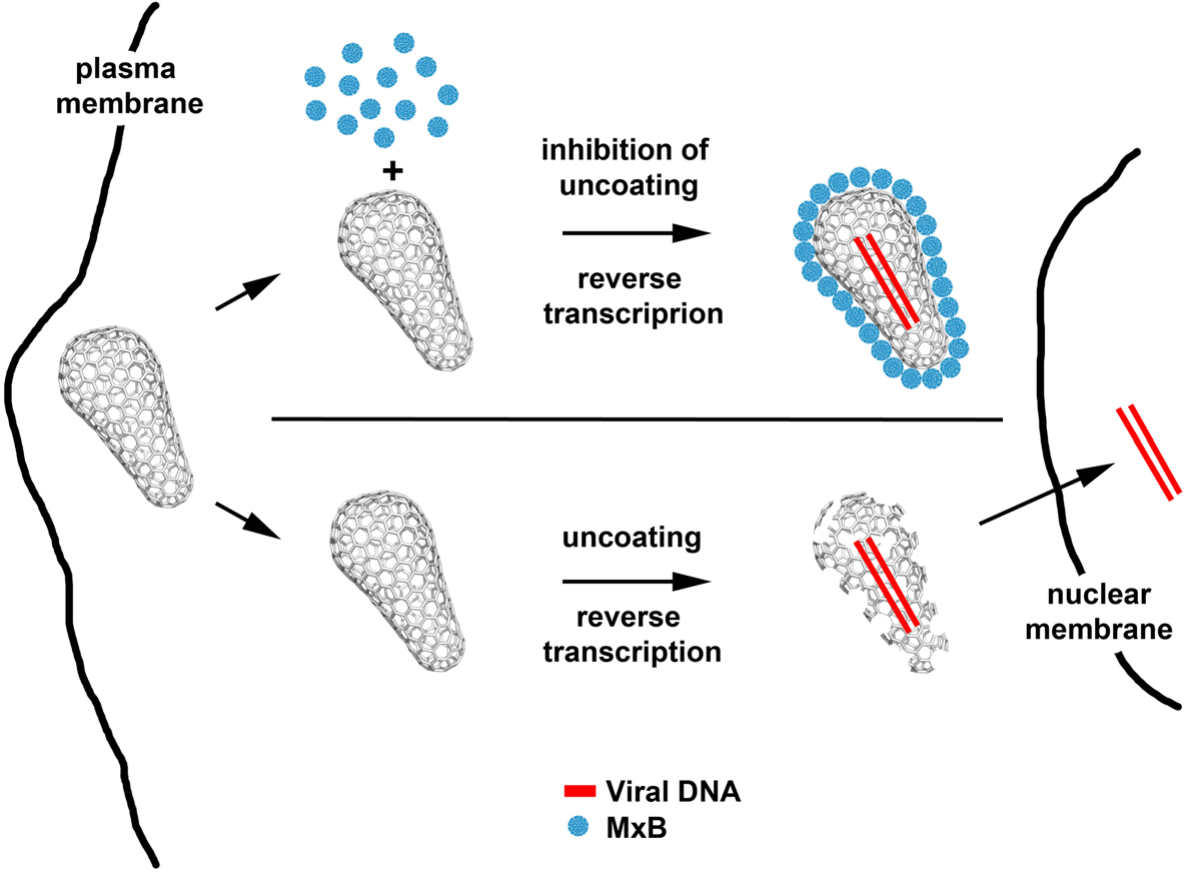
**MxB binds to the HIV-1 core and prevents the uncoating process of HIV-1.**

Overall our investigations suggested a model in which MxB encounters the HIV-1 core in the cytoplasm of the cell and prevents the uncoating process of HIV-1 (Figure 6). Our current understanding of uncoating suggests that HIV-1 reverse transcription occurs before or during the uncoating process of HIV-1 [25,47-50]. Interestingly, inhibition of uncoating by the use of proteasome inhibitors augments the occurrence of reverse transcription suggesting that reverse transcription can occur inside the HIV-1 core [23,32]. Similarly, inhibition of HIV-1 uncoating by NES-CPSF6 allows the occurrence of reverse transcription [33,34,51]. Therefore, it is feasible to propose that when MxB inhibits the uncoating process of HIV-1; it allows the occurrence of reverse transcription inside the HIV-1 core and traps the viral DNA. Trapping the viral DNA inside the core may prevent the transport of the viral DNA into the nucleus, in agreement with a block before nuclear import [21,22]. Similarly, the uncoating inhibitor NES-CPSF6 blocks HIV-1 replication before nuclear import [33,34,51]. An alternative possibility is that a block to nuclear import will promote the accumulation of HIV-1 cores in the cytosol resulting in an inhibition of the uncoating process of HIV-1.

## Conclusions

Overall, our work showed that MxB binds to the HIV-1 core and prevents the uncoating process of HIV-1. In addition, we demonstrated that MxB requires capsid binding and oligomerization for restriction.

## Methods

Protocols for each methodology used in this manuscript are explained in detail in Additional file 5.

### Infection with viruses expressing green fluorescent protein (GFP)

Recombinant HIV-1 expressing GFP was prepared as described [23]. Recombinant viruses were pseudotyped with the VSV-G glycoprotein. For infections, 3 × 10^4^ HeLa, U937 or Cf2Th cells seeded in 24-well plates were incubated at 37°C with virus for 24 h. Cells were washed and returned to culture for 48 h, and then GFP-positive cells were analyzed using a flow cytometer (Becton Dickinson).

### Binding of MxB variants to in vitro assembled HIV-1 capsid-nucleocapsid (CA-NC) complexes

The binding of different proteins to in vitro assembled HIV-1 CA-NC complexes that do or do not contain capsid changes was carried out as previously described [24,25]. Input and bound fractions were analyzed by Western blotting using the indicated antibodies.

### Fate of the capsid assay

The fate of the capsid assay was performed as previously described [25,28,29]. Cells infected for 16 hours were lysed using 15 strokes in a 7.0 ml Dounce homogenizer with pestle B. Cellular debris was cleared by centrifugation for 3 minutes at 3000 rpm. The cleared lysate was layered onto a 50% sucrose (weight: volume) cushion in 1× PBS and centrifuged at 125,000 × g for 2 hours at 4°C in a Beckman SW41 rotor. Input, soluble and pellet fractions were analyzed by Western blotting using the indicated antibodies.

### Creation of cells stably expressing wild type and mutant MxB proteins

Cell lines stably expressing wild type or mucbltant MxB proteins were created using the LPCX vector system (Clontech), as previously described [25,29,52]. The MxB proteins contained either an influenza hemagglutinin (HA) epitope tag or a FLAG epitope tag at the C terminus.

### MxB oligomerization assay

The ability of MxB to oligomerize was determined by measuring the interaction of an MxB-FLAG protein with an MxB-HA protein. Immunoprecipitates were analyzed by Western blotting using either anti-HA or anti-FLAG antibodies (Sigma).

## Additional files

Additional file 1:
**Restriction of HIV-1 and other viruses by MxB. (A)** HeLa cells stably expressing MxB (bottom panel) were challenged using increasing amounts of HIV-1-GFP. Forty-eight hours post-infection the percentage of GFP-positive cells was determined by flow cytometry. As a control, HeLa cells stably transduced with the empty vector LPCX were challenged with HIV-1. Similar results were obtained in three independent experiments and a representative experiment is shown. **(B)** U937 cells stably expressing MxB (bottom panel) were challenged with HIV-1-GFP and SIVmac-GFP. Forty-eight hours post-infection the percentage of GFP-positive cells was determined by flow cytometry. As a control, U937 cells stably transduced with the empty vector LPCX were challenged with HIV-1-GFP. Similar results were obtained in three independent experiments and a representative experiment is shown. **(C)** Cf2Th cells stably expressing MxB (bottom panel) were challenged with HIV-1-GFP, HIV-2-GFP, SIVmac-GFP, BIV-GFP, FIV-GFP, EAIV-GFP, N-MLV-GFP and BMLV- GFP. Forty-eight hours post-infection the percentage of GFP-positive cells was determined by flow cytometry. As a control, Cf2Th cells stably transduced with the empty vector LPCX were challenged with the indicated viruses. Similar results were obtained in three independent experiments and a representative experiment is shown. **(D)** The ability of MxB to bind in vitro assembled SIVmac (left panel) or HIV-2 (right panel) CA-NC complexes was measured, as described in experimental procedures. Bound fractions were analyzed using anti-HA, anti-p27 and anti-HIV-2-p24 antibodies. Similar results were obtained in three independent experiments and a representative experiment is shown. Additional file 2:
**Restriction of HIV-1 infection by MxB. (A)** Cf2Th cells stably expressing MxB or TRIM5α_rh_ were challenged with increasing amounts of HIV-1-GFP. Forty-eight hours post-infection the percentage of GFP-positive cells was determined by flow cytometry. As a control, Cf2Th cells stably transduced with the empty vector LPCX were challenged with HIV-1-GFP. **(B)** Cf2Th cells stably expressing MxB were challenged with increasing amounts of HIV-1, HIV-1-P90A, HIV-1-G89V or HIV-1-N57S expressing GFP as a reporter of infection. As a control, Cf2Th cells stably transduced with the empty vector LPCX were challenged with HIV-1-GFP. **(C)** Infectious units (IU) per ng of reverse transcriptase (RT) were measured for HIV-1, HIV-1-P90A, HIV- 1-G89V, and HIV-1-N57S. **(D)** The ability of CPSF6-FLAG to bind in vitro assembled HIV-1 CA-NC complexes bearing capsid changes P90A, G89V and N57S was measured as described in experimental procedures. As expected, CPSF6 binds in vitro assembled HIV-1 CA-NC complexes bearing changes P90A and G89V when compared to wild type (left panel). In agreement with findings suggesting that N57 is in the capsid region that interacts with CPSF6, we showed that CPSF6 poorly binds in vitro assembled HIV-1 CA-NC complexes bearing the change N57S (left panel). At the same time, the ability of TRIM5α_rh_ to bind in vitro assembled HIV-1 CA-NC complexes bearing the change N57S was tested (right panel). **(E)** In vitro assembled HIV-1 CA-NC complexes bearing the capsid changes P90A, G89V and N57S were negatively stained and analyzed by transmission electron microscopy. Representative fields are shown, and the scale bar corresponds to 100 nm. **(F)** Cf2Th cells stably expressing MxB, NES-CPSF6 or TRIM5αrh were challenged with increasing amounts of HIV-1-GFP. Forty-eight hours postinfection the percentage of GFP-positive cells was determined by flow cytometry. As a control, Cf2Th cells stably transduced with the empty vector LPCX were challenged with HIV-1-GFP. Additional file 3:
**Role of Cyclophilin A in MxB restriction. (A)** Cf2Th cells stably expressing MxB or TRIMCyp were challenged with increasing amounts of HIV-1-GFP in the presence of 10 μM cyclosporine A (CsA). Forty-eight hours post-infection the percentage of GFP-positive cells was determined by flow cytometry. As a control, Cf2Th cells stably transduced with the empty vector LPCX were challenged with HIV-1-GFP. Similar results were obtained in three independent experiments and a representative experiment is shown. **(B)** The ability of MxB and TRIMCyp to bind in vitro assembled HIV-1 CANC complexes in the presence of 10 μM CsA was measured, as described in Experimental Procedures. Similar results were obtained in three independent experiments and a representative experiment is shown. **(C)** The ability of MxB to recruit endogenously expressed cyclophilin A to in vitro assembled HIV-1 CA-NC complexes was measured by performing a capsid binding assay, as describe in experimental procedures. Input and Bound fractions were analyzed by Western blotting using antibodies against FLAG, cyclophilin A and p24. Similar results were obtained in three independent experiments and a representative experiment is shown. Additional file 4:
**Binding of MxB variants to in vitro assembled HIV-1 CANC complexes.** The ability of wild type and mutant MxB proteins to bind in vitro assembled HIV-1 CA-NC complexes was measured, as described in experimental procedures. Input and bound fractions were analyzed by Western blotting using anti-FLAG and anti-p24 antibodies. Similar results were obtained in three independent experiments and a representative experiment is shown. Additional file 5:
**Detailed explanation of used methodologies.**
