# Supplementary material for: MxB binds to the HIV-1 core and prevents the uncoating process of HIV-1
Source: Retrovirology. 2014 Aug 14;11:68. doi: 10.1186/s12977-014-0068-x (PMC4145229; doi:10.1186/s12977-014-0068-x)
Supplement: Additional file 1: — Restriction of HIV-1 and other viruses by MxB. (A) HeLa cells stably expressing MxB (bottom panel) were challenged using increasing amounts of HIV-1-GFP. Forty-eight hours post-infection the percentage of GFP-positive cells was determined by flow cytometry. As a control, HeLa cells stably transduced with the empty vector LPCX were challenged with HIV-1. Similar results were obtained in three independent experiments and a representative experiment is shown. (B) U937 cells stably expressing MxB (bottom panel) were challenged with HIV-1-GFP and SIVmac-GFP. Forty-eight hours post-infection the percentage of GFP-positive cells was determined by flow cytometry. As a control, U937 cells stably transduced with the empty vector LPCX were challenged with HIV-1-GFP. Similar results were obtained in three independent experiments and a representative experiment is shown. (C) Cf2Th cells stably expressing MxB (bottom panel) were challenged with HIV-1-GFP, HIV-2-GFP, SIVmac-GFP, BIV-GFP, FIV-GFP, EAIV-GFP, N-MLV-GFP and BMLV- GFP. Forty-eight hours post-infection the percentage of GFP-positive cells was determined by flow cytometry. As a control, Cf2Th cells stably transduced with the empty vector LPCX were challenged with the indicated viruses. Similar results were obtained in three independent experiments and a representative experiment is shown. (D) The ability of MxB to bind in vitro assembled SIVmac (left panel) or HIV-2 (right panel) CA-NC complexes was measured, as described in experimental procedures. Bound fractions were analyzed using anti-HA, anti-p27 and anti-HIV-2-p24 antibodies. Similar results were obtained in three independent experiments and a representative experiment is shown. [file 12977_2014_68_MOESM1_ESM.pdf]

Additional file 1. Restriction of HIV-1 and other viruses by MxB.

(A) HeLa cells stably expressing MxB (bottom panel) were challenged using increasing amounts of HIV-1-GFP. Forty-eight hours post-infection the percentage of GFP-positive cells was determined by flow cytometry. As a control, HeLa cells stably transduced with the empty vector LPCX were challenged with HIV-1. Similar results were obtained in three independent experiments and a representative experiment is shown. **(B)** U937 cells stably expressing MxB (bottom panel) were challenged with HIV-1-GFP and SIV_{mac}-GFP. Forty-eight hours post-infection the percentage of GFP-positive cells was determined by flow cytometry. As a control, U937 cells stably transduced with the empty vector LPCX were challenged with HIV-1-GFP. Similar results were obtained in three independent experiments and a representative experiment is shown. **(C)** Cf2Th cells stably expressing MxB (bottom panel) were challenged with HIV-1-GFP, HIV-2-GFP, SIV_{mac}-GFP, BIV-GFP, FIV-GFP, EAIV-GFP, N-MLV-GFP and B-MLV-GFP. Forty-eight hours post-infection the percentage of GFP-positive cells was determined by flow cytometry. As a control, Cf2Th cells stably transduced with the empty vector LPCX were challenged with the indicated viruses. Similar results were obtained in three independent experiments and a representative experiment is shown. **(D)** The ability of MxB to bind in vitro assembled SIV_{mac} (left panel) or HIV-2 (right panel) CA-NC complexes was measured, as described in experimental procedures. Bound fractions were analyzed using anti-HA, anti-p27 and anti-HIV-2-p24 antibodies. Similar results were obtained in three independent experiments and a representative experiment is shown.

A

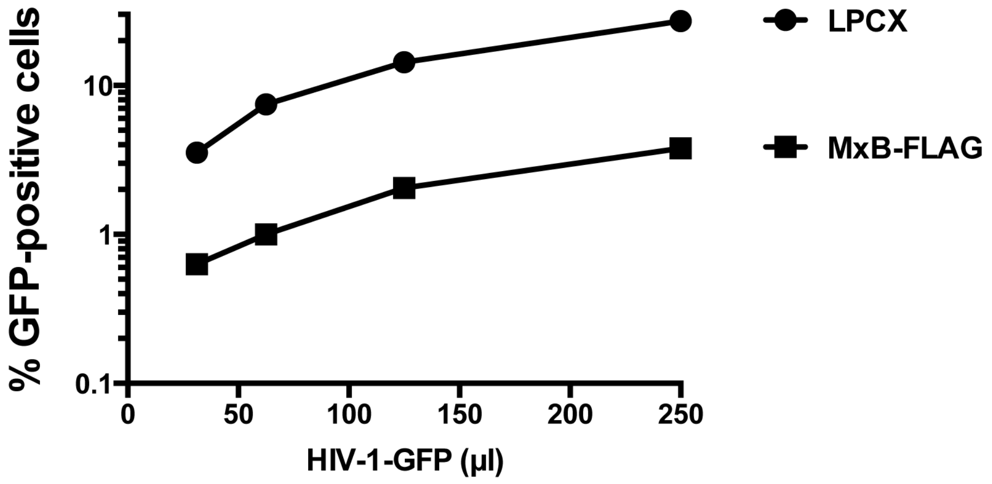

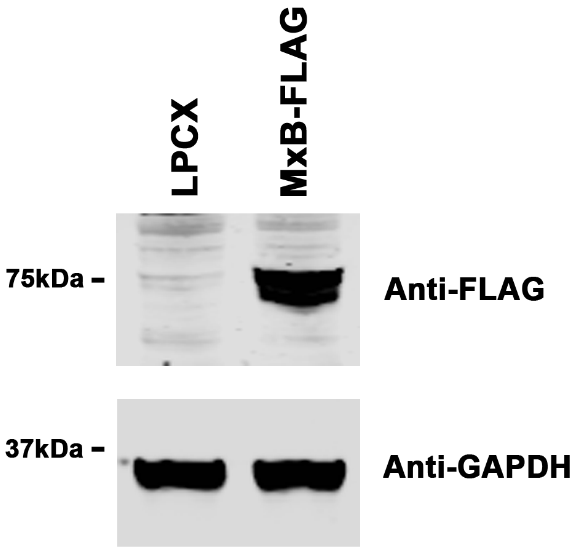

Additional file 1

B

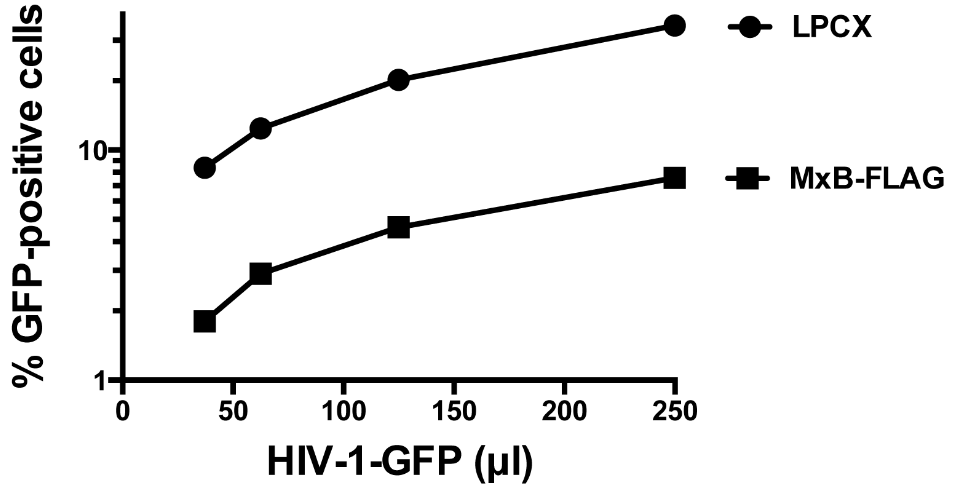

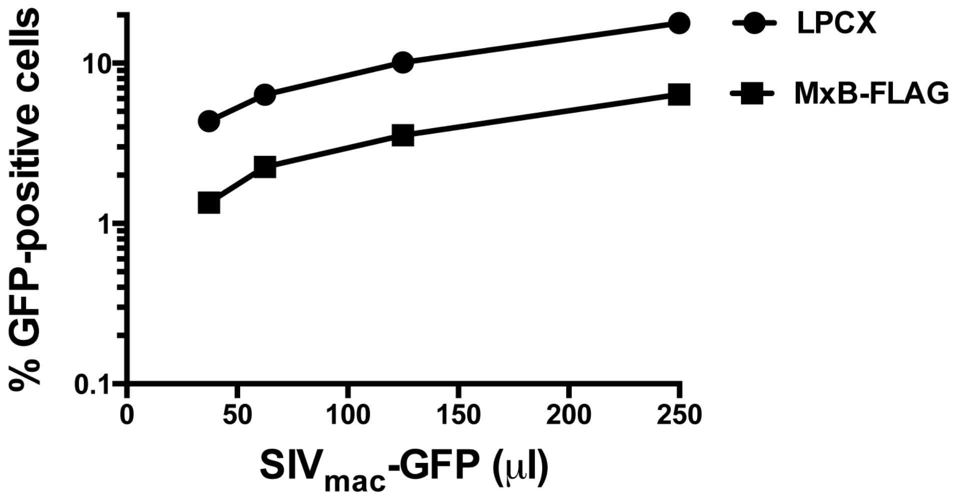

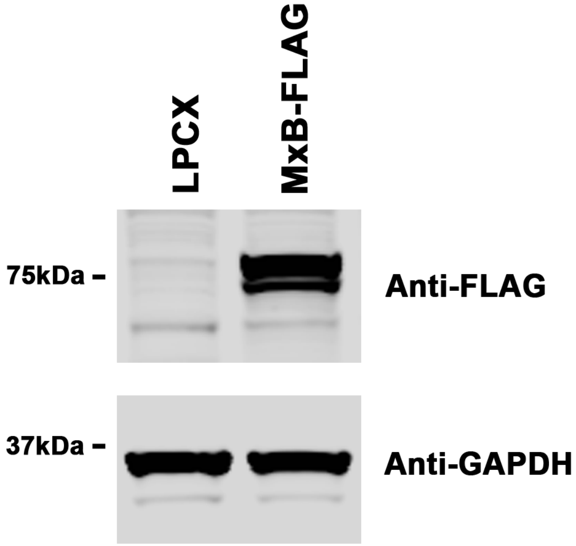

Additional file 1

C

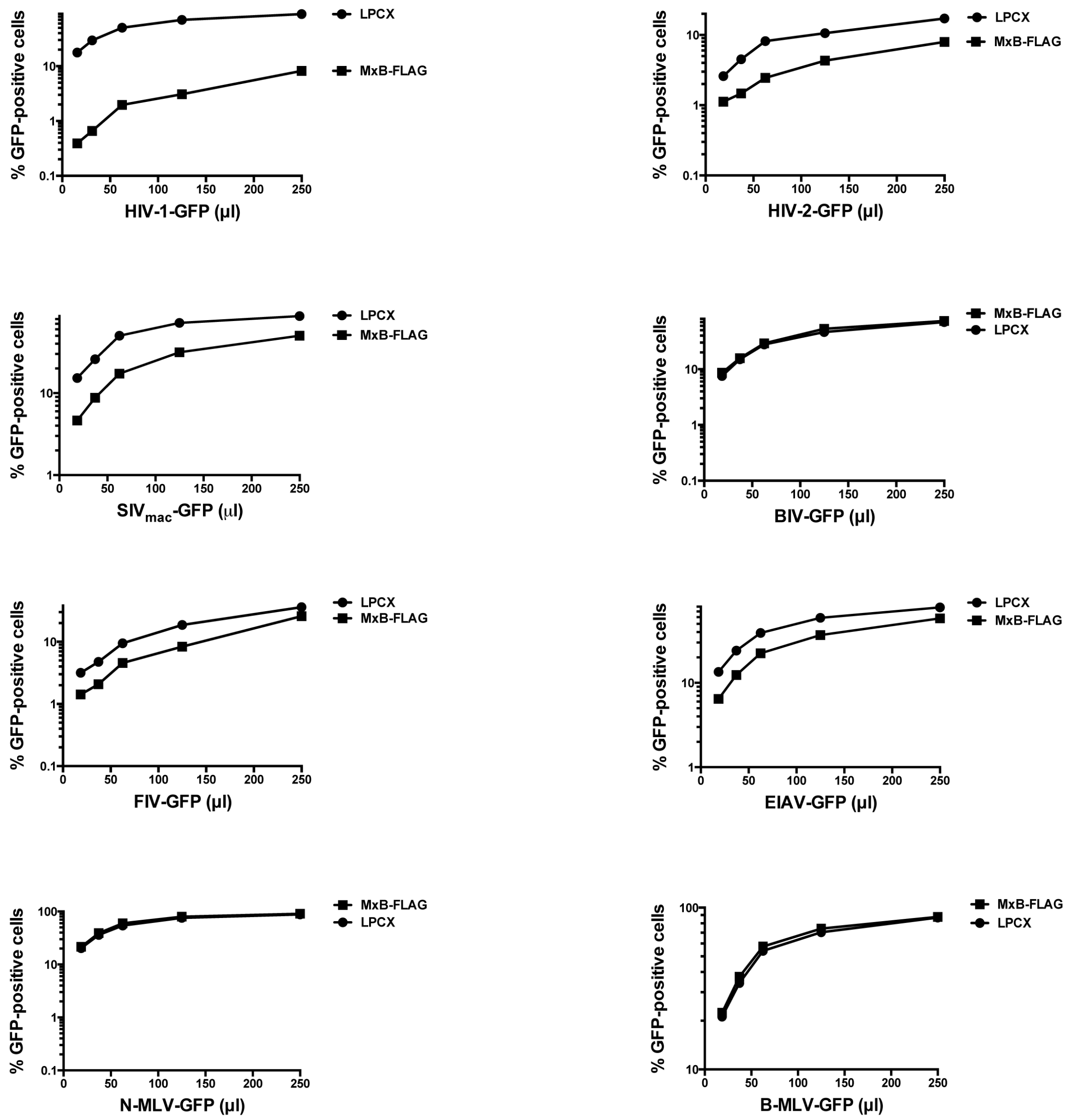

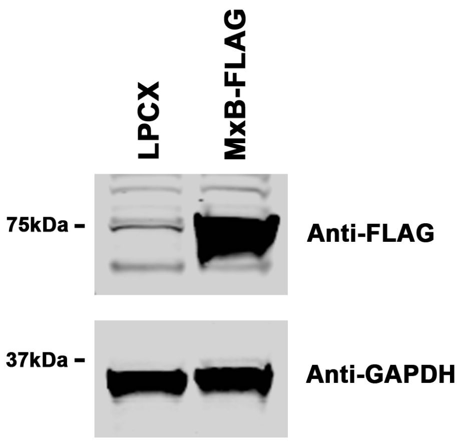

Additional file 1

Additional file 1

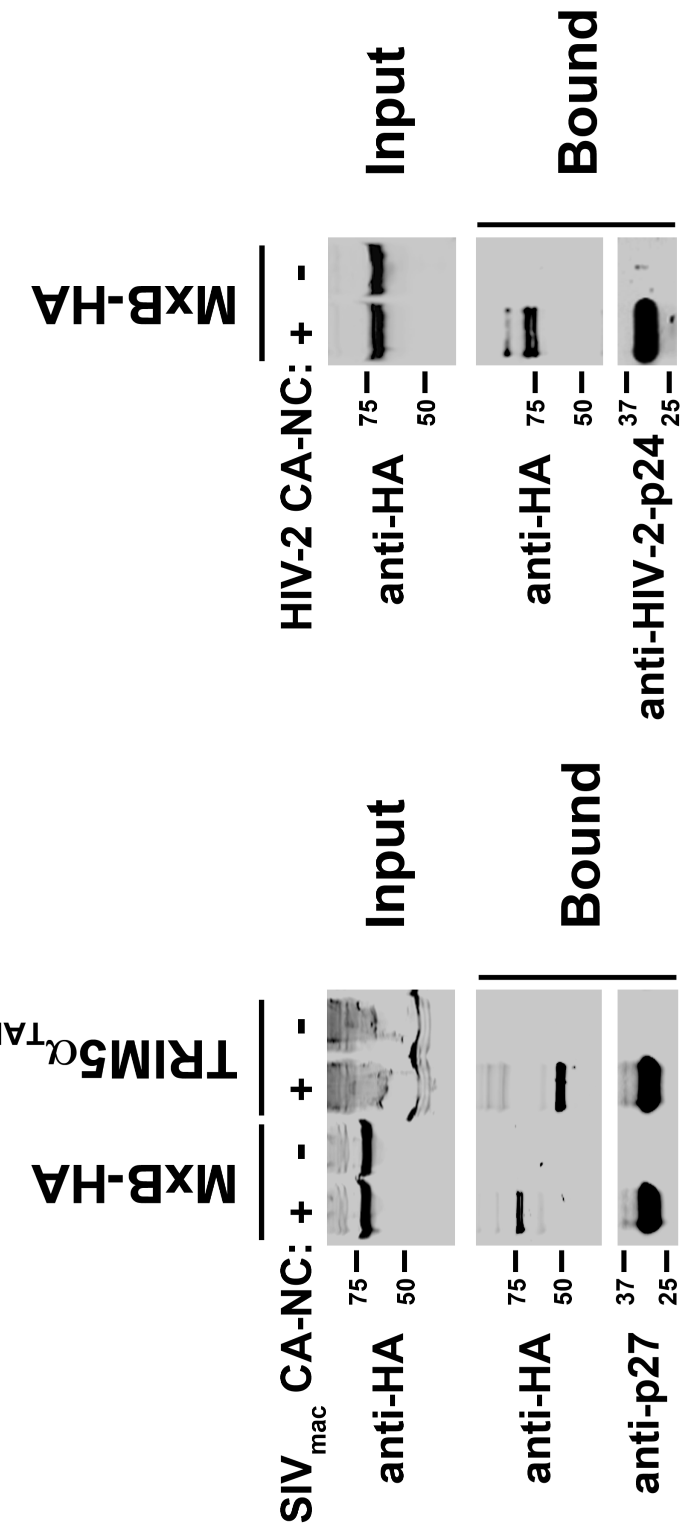

D
