# Supplementary material for: MxB binds to the HIV-1 core and prevents the uncoating process of HIV-1
Source: Retrovirology. 2014 Aug 14;11:68. doi: 10.1186/s12977-014-0068-x (PMC4145229; doi:10.1186/s12977-014-0068-x)
Supplement: Additional file 2: — Restriction of HIV-1 infection by MxB. (A) Cf2Th cells stably expressing MxB or TRIM5αrh were challenged with increasing amounts of HIV-1-GFP. Forty-eight hours post-infection the percentage of GFP-positive cells was determined by flow cytometry. As a control, Cf2Th cells stably transduced with the empty vector LPCX were challenged with HIV-1-GFP. (B) Cf2Th cells stably expressing MxB were challenged with increasing amounts of HIV-1, HIV-1-P90A, HIV-1-G89V or HIV-1-N57S expressing GFP as a reporter of infection. As a control, Cf2Th cells stably transduced with the empty vector LPCX were challenged with HIV-1-GFP. (C) Infectious units (IU) per ng of reverse transcriptase (RT) were measured for HIV-1, HIV-1-P90A, HIV- 1-G89V, and HIV-1-N57S. (D) The ability of CPSF6-FLAG to bind in vitro assembled HIV-1 CA-NC complexes bearing capsid changes P90A, G89V and N57S was measured as described in experimental procedures. As expected, CPSF6 binds in vitro assembled HIV-1 CA-NC complexes bearing changes P90A and G89V when compared to wild type (left panel). In agreement with findings suggesting that N57 is in the capsid region that interacts with CPSF6, we showed that CPSF6 poorly binds in vitro assembled HIV-1 CA-NC complexes bearing the change N57S (left panel). At the same time, the ability of TRIM5αrh to bind in vitro assembled HIV-1 CA-NC complexes bearing the change N57S was tested (right panel). (E) In vitro assembled HIV-1 CA-NC complexes bearing the capsid changes P90A, G89V and N57S were negatively stained and analyzed by transmission electron microscopy. Representative fields are shown, and the scale bar corresponds to 100 nm. (F) Cf2Th cells stably expressing MxB, NES-CPSF6 or TRIM5αrh were challenged with increasing amounts of HIV-1-GFP. Forty-eight hours postinfection the percentage of GFP-positive cells was determined by flow cytometry. As a control, Cf2Th cells stably transduced with the empty vector LPCX were challenged with HIV-1-GFP. [file 12977_2014_68_MOESM2_ESM.pdf]

Additional file 2. Restriction of HIV-1 infection by MxB.

(A) Cf2Th cells stably expressing MxB or TRIM5α_{rh} were challenged with increasing amounts of HIV-1-GFP. Forty-eight hours post-infection the percentage of GFP-positive cells was determined by flow cytometry. As a control, Cf2Th cells stably transduced with the empty vector LPCX were challenged with HIV-1-GFP. Similar results were obtained in three independent experiments and a representative experiment is shown. **(B)** Cf2Th cells stably expressing MxB were challenged with increasing amounts of HIV-1, HIV-1-P90A, HIV-1-G89V or HIV-1-N57S expressing GFP as a reporter of infection. Forty-eight hours post-infection the percentage of GFP-positive cells was determined by flow cytometry. As a control, Cf2Th cells stably transduced with the empty vector LPCX were challenged with HIV-1-GFP. Similar results were obtained in three independent experiments and a representative experiment is shown. **(C)** Infectious units (IU) per ng of reverse transcriptase (RT) were measured for HIV-1, HIV-1-P90A, HIV-1-G89V, and HIV-1-N57S. Experiments were performed in triplicate and a representative experiment is shown. **(D)** The ability of CPSF6-FLAG to bind in vitro assembled HIV-1 CA-NC complexes bearing capsid changes P90A, G89V and N57S was measured as described in experimental procedures. As expected, CPSF6 binds in vitro assembled HIV-1 CA-NC complexes bearing changes P90A and G89V when compared to wild type (left panel). In agreement with findings suggesting that N57 is in the capsid region that interacts with CPSF6 (Price et al., 2012), we showed that CPSF6 poorly binds in vitro assembled HIV-1 CA-NC complexes bearing the change N57S (left panel). At the same time, the ability of

TRIM5α_{rh} to bind in vitro assembled HIV-1 CA-NC complexes bearing the change N57S was tested (right panel). Similar results were obtained in three independent experiments and a representative experiment is shown. **(E)** In vitro assembled HIV-1 CA-NC complexes bearing the capsid changes P90A, G89V and N57S were negatively stained and analyzed by transmission electron microscopy. Representative fields are shown, and the scale bar corresponds to 100 nm. **(F)** Cf2Th cells stably expressing MxB, NES-CPSF6 or TRIM5α_{rh} were challenged with increasing amounts of HIV-1-GFP. Forty-eight hours post-infection the percentage of GFP-positive cells was determined by flow cytometry. As a control, Cf2Th cells stably transduced with the empty vector LPCX were challenged with HIV-1-GFP. Similar results were obtained in three independent experiments and a representative experiment is shown.

A

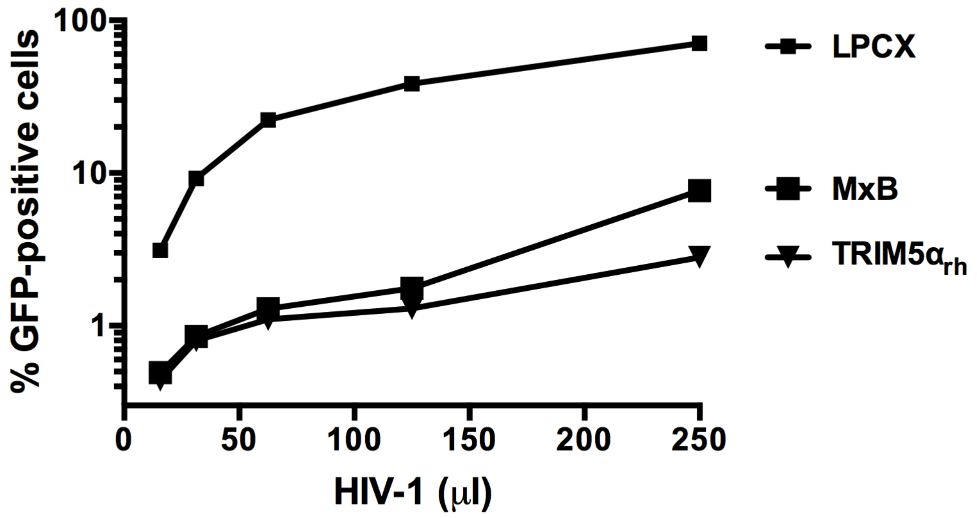

Additional file 2

B

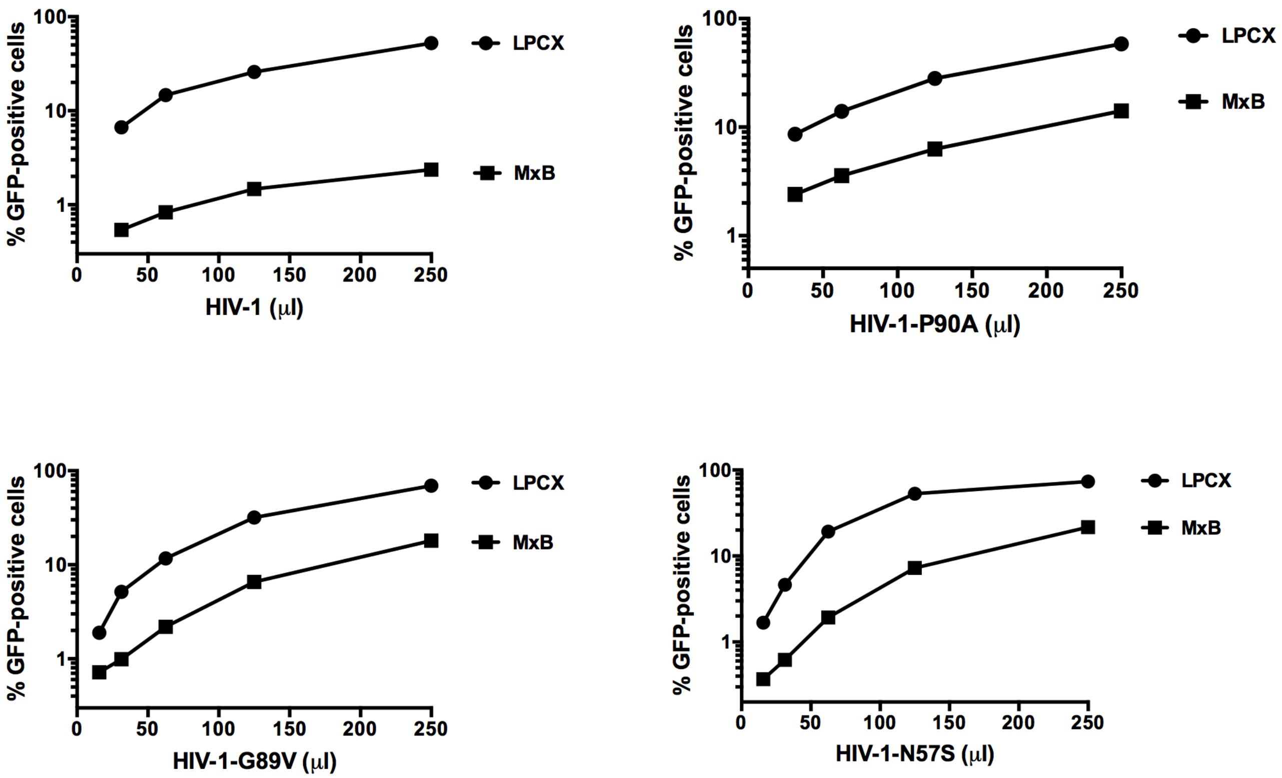

Additional file 2

C

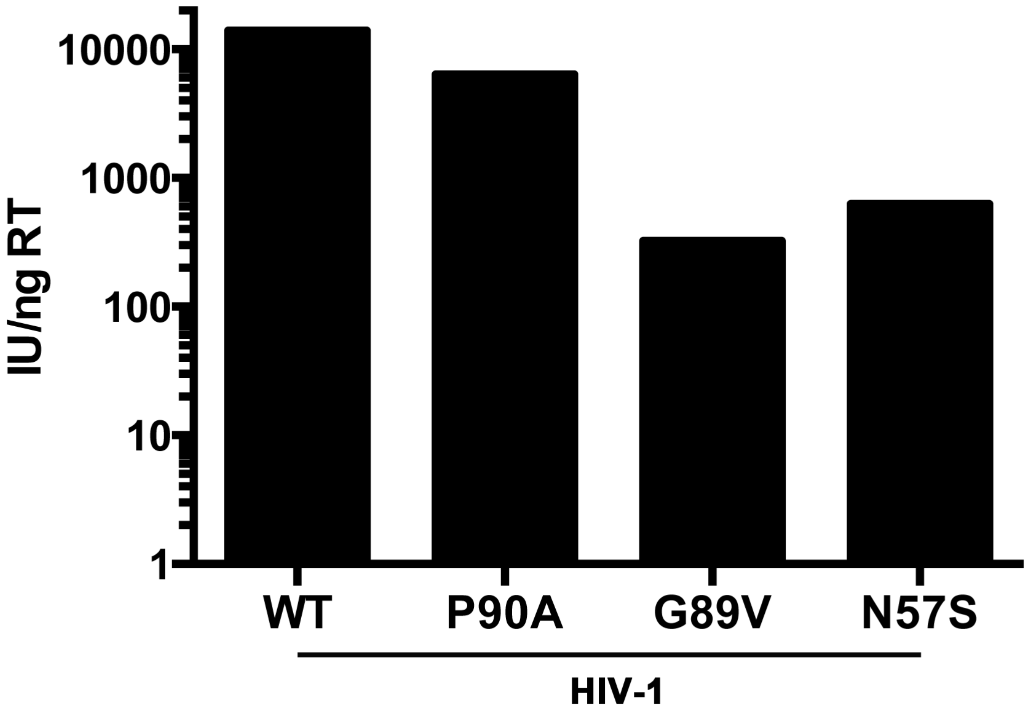

Additional file 2

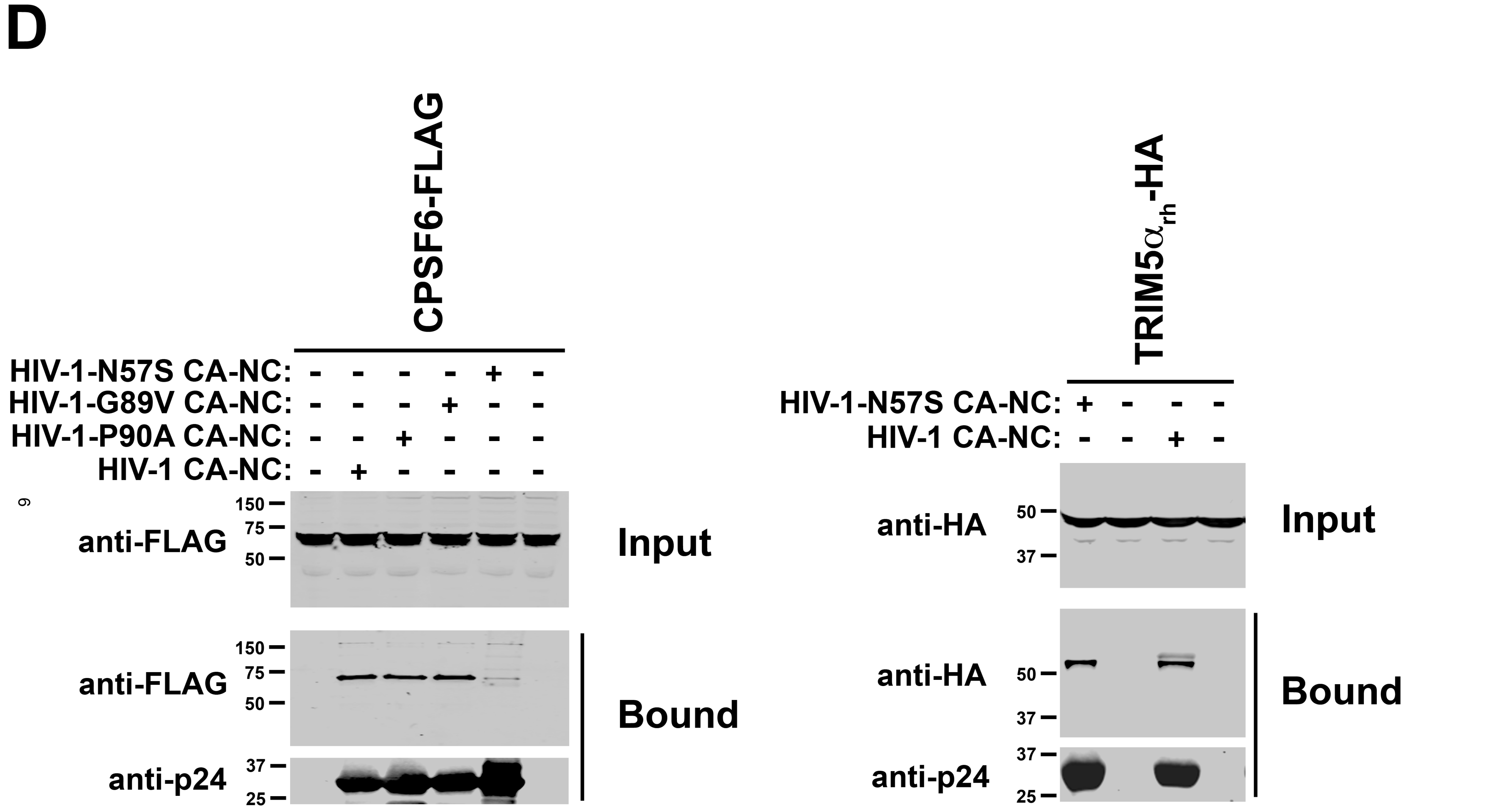

Additional file 2

E

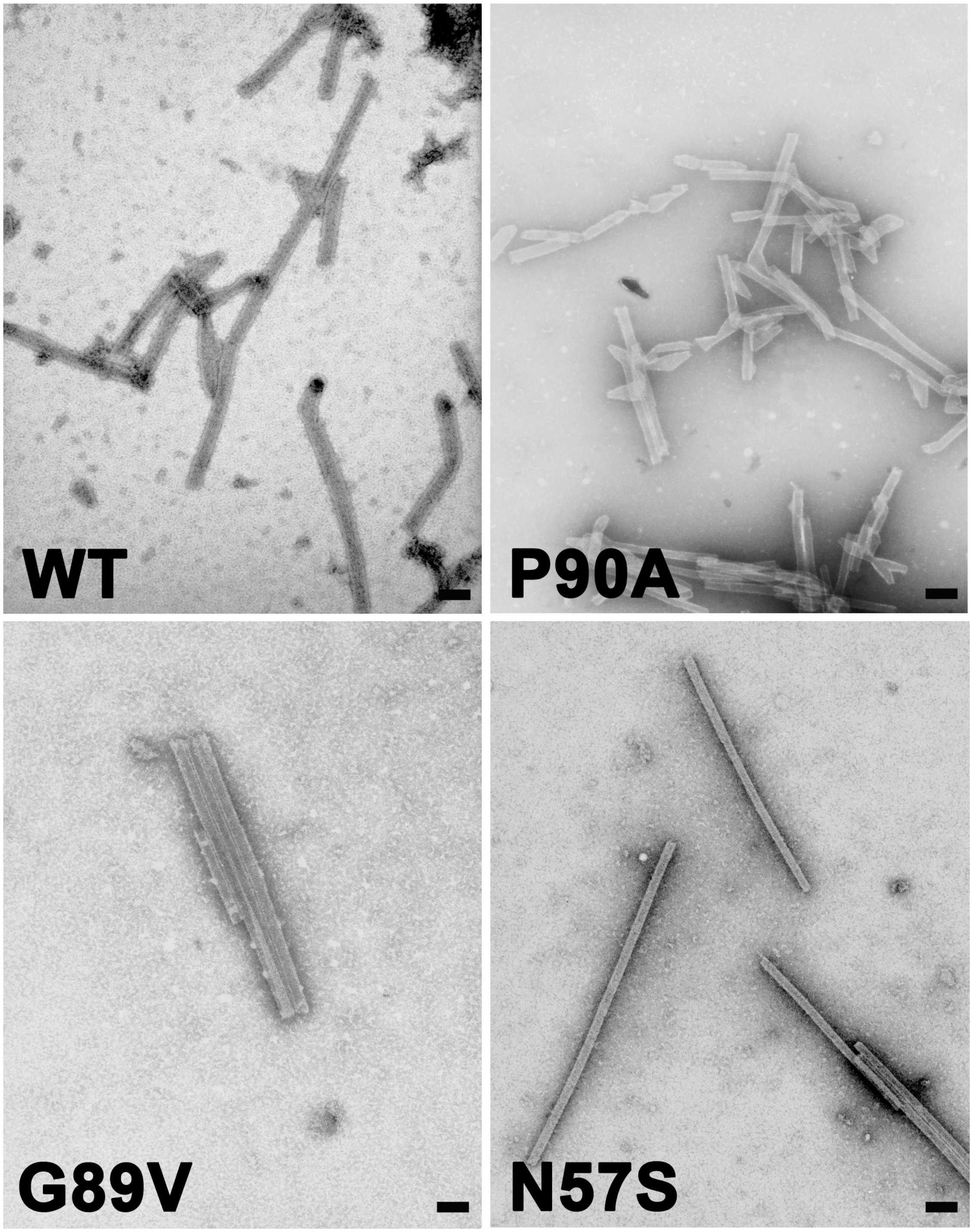

Additional file 2

F

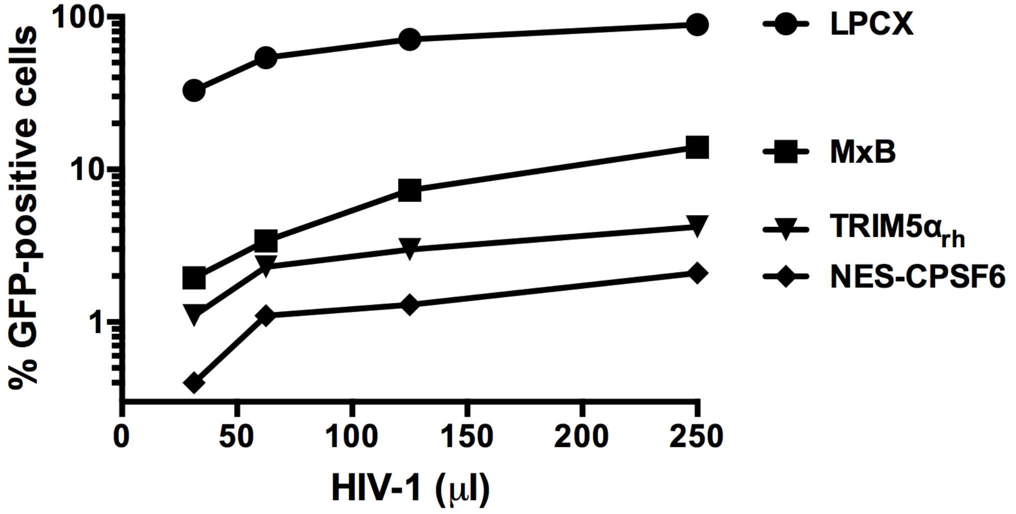

Additional file 2
