# Supplementary material for: MxB binds to the HIV-1 core and prevents the uncoating process of HIV-1
Source: Retrovirology. 2014 Aug 14;11:68. doi: 10.1186/s12977-014-0068-x (PMC4145229; doi:10.1186/s12977-014-0068-x)
Supplement: Additional file 3: — Role of Cyclophilin A in MxB restriction. (A) Cf2Th cells stably expressing MxB or TRIMCyp were challenged with increasing amounts of HIV-1-GFP in the presence of 10 μM cyclosporine A (CsA). Forty-eight hours post-infection the percentage of GFP-positive cells was determined by flow cytometry. As a control, Cf2Th cells stably transduced with the empty vector LPCX were challenged with HIV-1-GFP. Similar results were obtained in three independent experiments and a representative experiment is shown. (B) The ability of MxB and TRIMCyp to bind in vitro assembled HIV-1 CANC complexes in the presence of 10 μM CsA was measured, as described in Experimental Procedures. Similar results were obtained in three independent experiments and a representative experiment is shown. (C) The ability of MxB to recruit endogenously expressed cyclophilin A to in vitro assembled HIV-1 CA-NC complexes was measured by performing a capsid binding assay, as describe in experimental procedures. Input and Bound fractions were analyzed by Western blotting using antibodies against FLAG, cyclophilin A and p24. Similar results were obtained in three independent experiments and a representative experiment is shown. [file 12977_2014_68_MOESM3_ESM.pdf]

Additional file 3. Role of Cyclophilin A in MxB restriction.

(A) Cf2Th cells stably expressing MxB or TRIMCyp were challenged with increasing amounts of HIV-1-GFP in the presence of 10 μM cyclosporine A (CsA). Forty-eight hours post-infection the percentage of GFP-positive cells was determined by flow cytometry. As a control, Cf2Th cells stably transduced with the empty vector LPCX were challenged with HIV-1-GFP. Similar results were obtained in three independent experiments and a representative experiment is shown. **(B)** The ability of MxB and TRIMCyp to bind in vitro assembled HIV-1 CA-NC complexes in the presence of 10 μM CsA was measured, as described in Experimental Procedures. Similar results were obtained in three independent experiments and a representative experiment is shown. **(C)** The ability of MxB to recruit endogenously expressed cyclophilin A to in vitro assembled HIV-1 CA-NC complexes was measured by performing a capsid binding assay, as describe in experimental procedures. Input and Bound fractions were analyzed by Western blotting using antibodies against FLAG, cyclophilin A and p24. Similar results were obtained in three independent experiments and a representative experiment is shown.

A

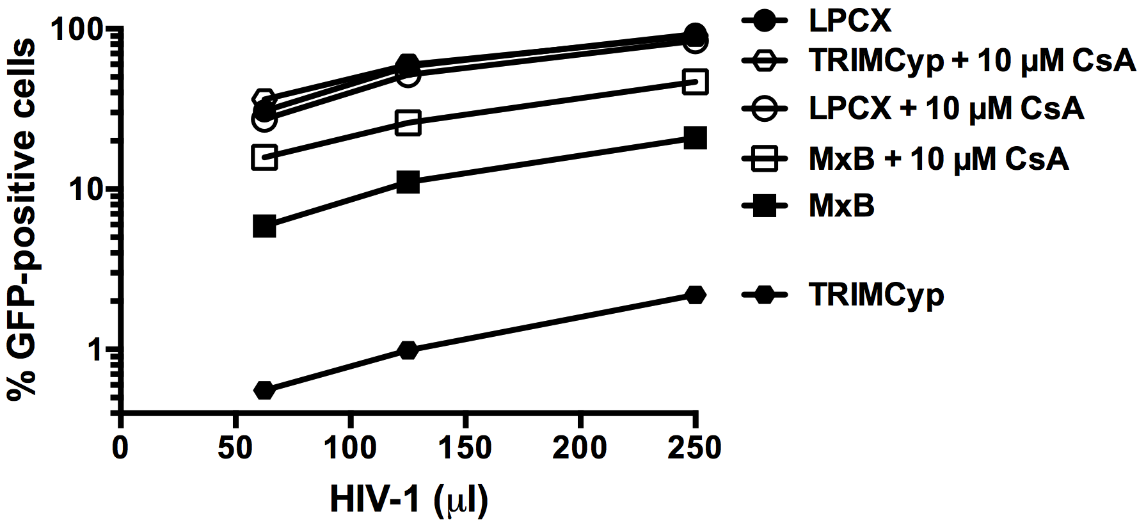

Additional file 3

B

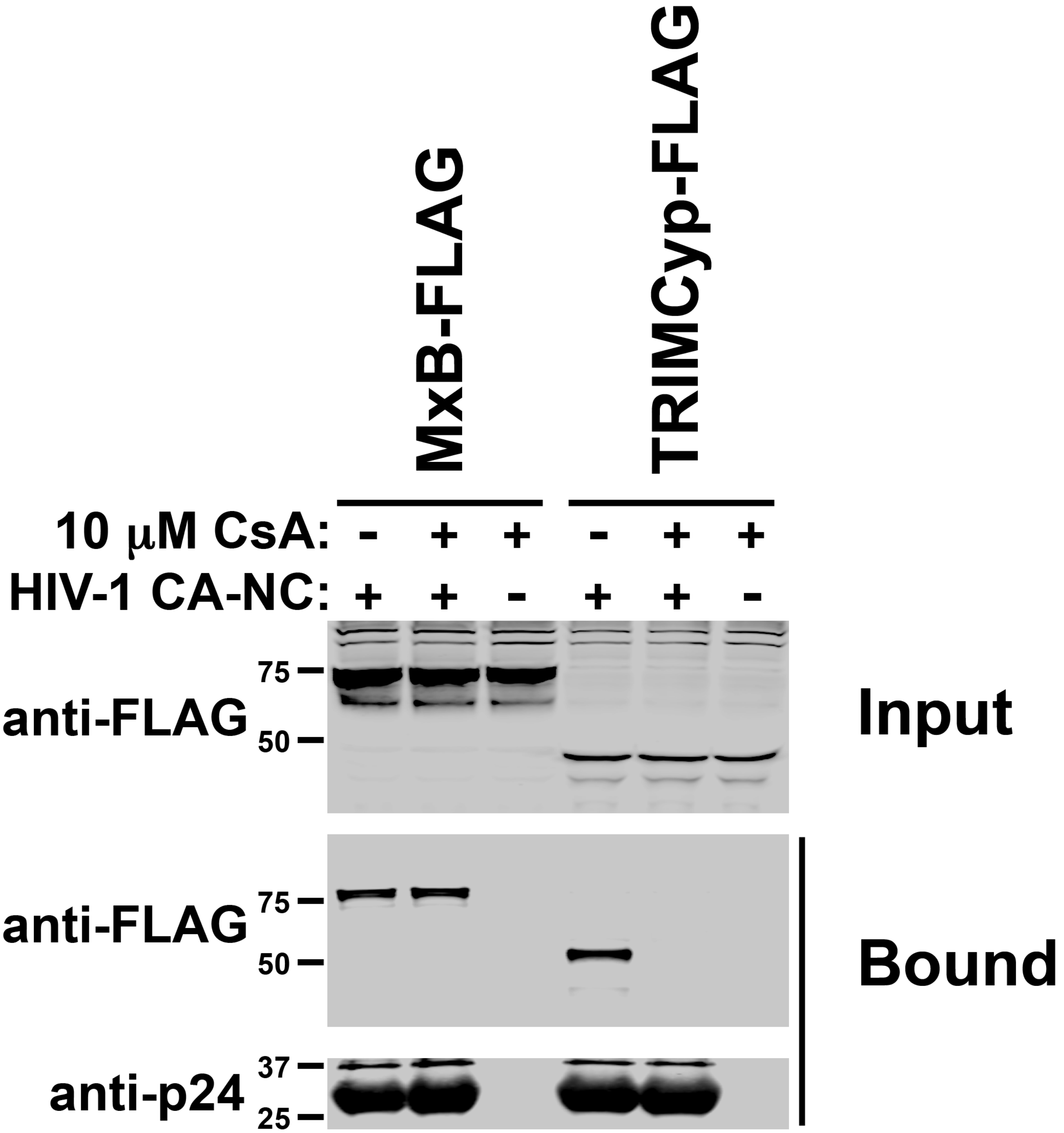

Additional file 3

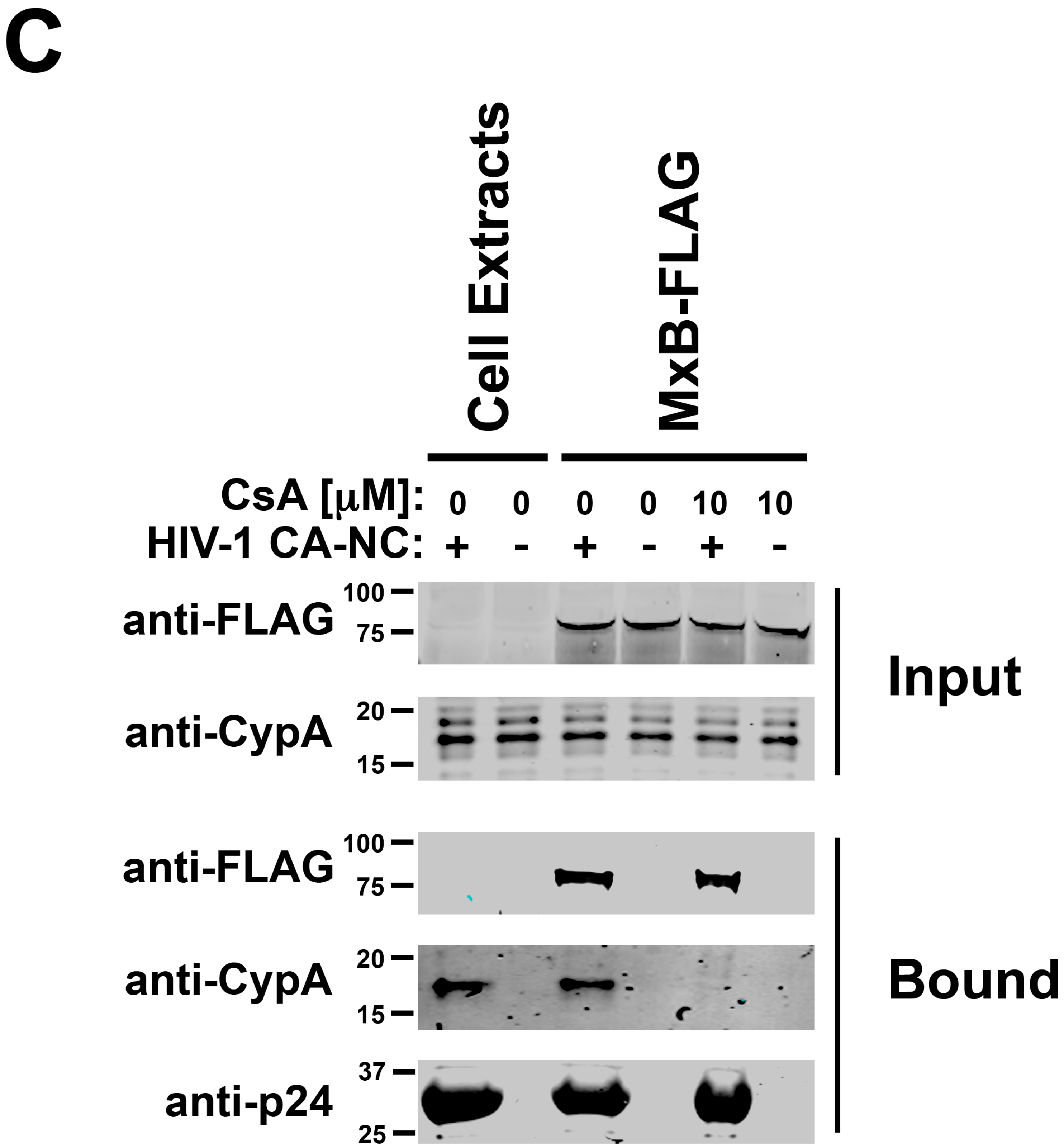

Additional file 3
