# Supplementary material for: MxB binds to the HIV-1 core and prevents the uncoating process of HIV-1
Source: Retrovirology. 2014 Aug 14;11:68. doi: 10.1186/s12977-014-0068-x (PMC4145229; doi:10.1186/s12977-014-0068-x)
Supplement: Additional file 4: — Binding of MxB variants to in vitro assembled HIV-1 CANC complexes. The ability of wild type and mutant MxB proteins to bind in vitro assembled HIV-1 CA-NC complexes was measured, as described in experimental procedures. Input and bound fractions were analyzed by Western blotting using anti-FLAG and anti-p24 antibodies. Similar results were obtained in three independent experiments and a representative experiment is shown. [file 12977_2014_68_MOESM4_ESM.pdf]

Additional file 4. Binding of MxB variants to in vitro assembled HIV-1 CA-NC complexes. The ability of wild type and mutant MxB proteins to bind in vitro assembled HIV-1 CA-NC complexes was measured, as described in experimental procedures. Input and bound fractions were analyzed by Western blotting using anti-FLAG and anti-p24 antibodies. Similar results were obtained in three independent experiments and a representative experiment is shown.

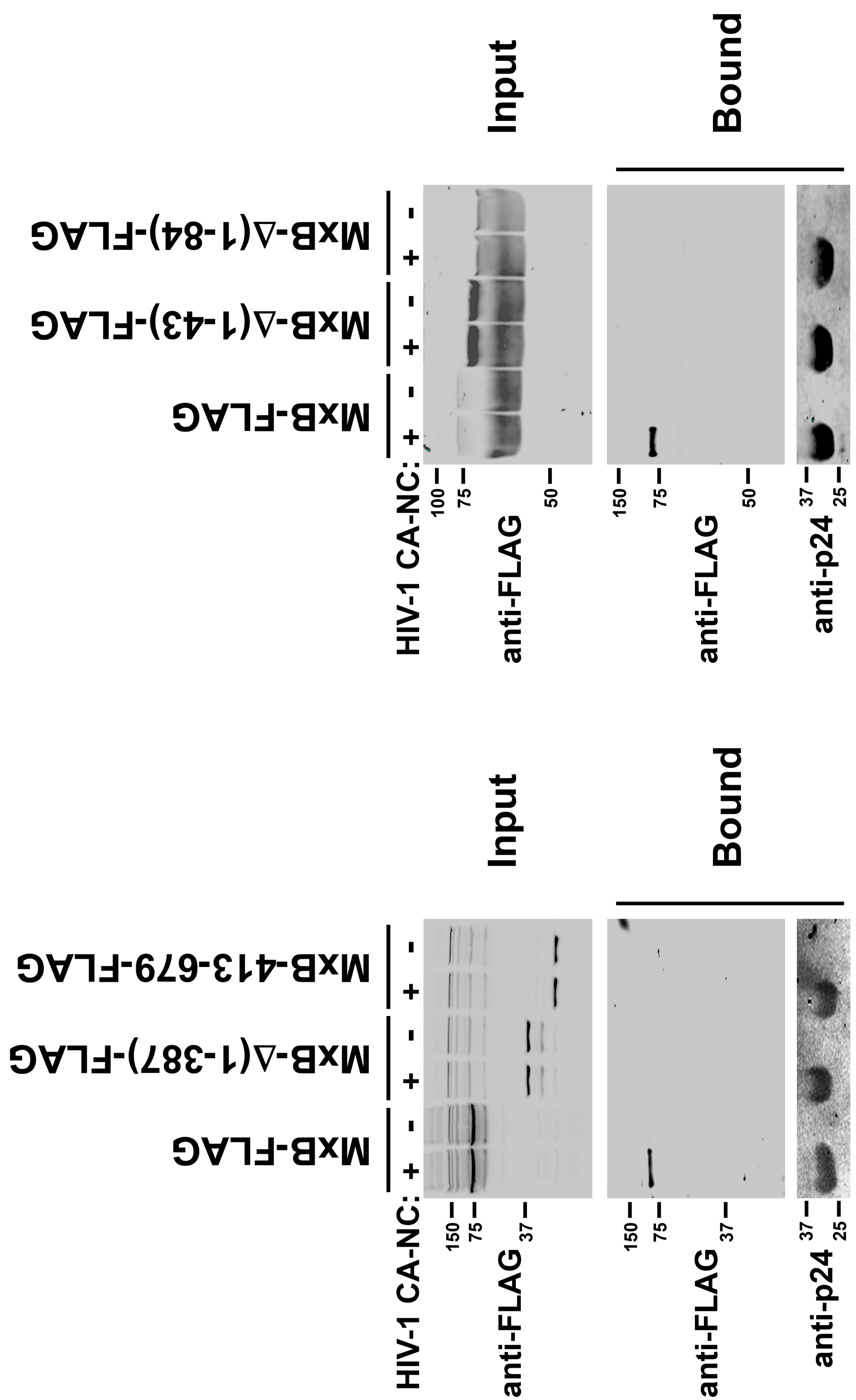

Additional file 4
