# Supplementary material for: MxB binds to the HIV-1 core and prevents the uncoating process of HIV-1
Source: Retrovirology. 2014 Aug 14;11:68. doi: 10.1186/s12977-014-0068-x (PMC4145229; doi:10.1186/s12977-014-0068-x)
Supplement: Additional file 5: — Detailed explanation of used methodologies. [file 12977_2014_68_MOESM5_ESM.doc]

**Additional File 5**

**Reagents**

The stock solution of cyclosporine (Sigma; PHR1092; 500 mg) was prepared at

10 mM in ethanol. The CAI peptide (amino acid sequence, ITFEDLLDYYGP) was

synthesized by Genescript (95% purity), and stock solutions were prepared at 10

mM in dimethyl sulfoxide (DMSO). The stock solution of PF74 (PF-3450074)

was prepared at 100 mM in DMSO. The stock solution of BI-2 was prepared at

20 mM in DMSO. The stock solution of CPIPB (4-{2-[3-(3-clorophenyl)-1Hpyrazol-

4-yl]-1-[3-(1H-imidazol-1-yl-propyl]-1H-benzimidazol-5-yl}benzoic acid)

was prepared at 20 mM in water. CAP-1 (N-(3-chloro-4-methylphenyl)-N′-{2-[({5-

[(dimethylamino)-methyl]-2-furyl}-methyl)-sulfanyl] ethyl}-urea) was purchased

from Maybridge (HTS 02911). The stock solution of CAP-1 was prepared at 20

mM in DMSO. Antibodies against human Cyclophilin A were obtained from Cell

Signaling (Cat#2175).

**Infection with viruses expressing green fluorescent protein (GFP)**

Recombinant HIV-1 expressing GFP was prepared as described (Diaz-Griffero et

al., 2008). Recombinant viruses were pseudotyped with the VSV-G glycoprotein.

For infections, 3×10^4^ HeLa, U937 or Cf2Th cells seeded in 24-well plates were

incubated at 37 °C with virus for 24 h. Cells were washed and returned to culture

for 48 h, and then GFP-posiitve cells were analyzed using a flow cytometer

(Becton Dickinson).

**Binding of MxB variants to in vitro assembled HIV-1 capsidnucleocapsid(**

**CA-NC) complexes**

293T cells were transfected with plasmids expressing wild-type or mutant MxB

proteins. Forty-eight hours after transfection, cell lysates were prepared as

follows: previously washed cells were resuspended in hypotonic lysis buffer

(10 mM Tris, pH 7.4, 1.5 mM MgCl2, 10 mM KCl, and 0.5 mM DTT). The cell

suspension was frozen and thawed, and incubated on ice for 10 min. Afterwards,

the lysate was centrifuged at maximum speed in a refrigerated Eppendorf micro

centrifuge (~14,000*g*) for 5 min. The supernatant was supplemented with 1/10

volume of 10× PBS and then used in the binding assay. In some cases, samples

containing the TRIM5αrh variants were diluted with extracts prepared in parallel

from untransfected cells. To test binding, 5 μl of CA-NC particles assembled in

vitro were incubated with 200 μl of cell lysate at room temperature for 1 h. A

fraction of this mixture was stored (input). The mixture was spun through a 70%

sucrose cushion (70% sucrose, 1× PBS and 0.5 mM DTT) at 100,000*g* in an

SW55 rotor (Beckman) for 1 h at 4 °C. After centrifugation, the supernatant was

carefully removed and the pellet resuspended in 1× SDS-PAGE loading buffer

(pellet). The level of MxB proteins was determined by Western blotting with an

anti-HA or anti-FLAG antibody as described above. The level of HIV-1 CA–NC

protein in the pellet was assessed by Western blotting with an anti-p24 CA

antibody.

**Fate of the capsid assay**

The fate of the capsid assay was performed as previously described(Stremlau et

al., 2006; Yang et al., 2014). HIV-1 virus-like particles (VLPs) were produced by

calcium phosphate co-transfection of plasmids containing the following genes:

HIV-1 gag-pol, VSV-G envelope and rev protein at a weight:ratio 15:3:1. Stably

transduced Cf2Th (1.5 × 10^6^) cells expressing the indicated proteins were

seeded in 80cm2 flasks. The following day, the cells were incubated with 5-10 ml

(approximately 2.5- 5.0 × 10^5^ reverse transcriptase units) of HIV-1 at 4°C for 30

minutes to allow viral attachment to the cells. The cells were then shifted to 37°C

until they were harvested 16 hours post-infection. Cells were washed three times

using ice-cold PBS and detached by treatment with 1.0 ml of pronase (7.0mg/ml

in DMEM) for 5 minutes at 25°C. The cells were then washed three times with

PBS. The cells were resuspended in 2.5 ml hypotonic lysis buffer (10 mM Tris-

HCl, pH 8.0, 10 mM KCl, 1 mM EDTA and one complete protease inhibitor tablet)

and incubated on ice for 15 minutes. The cells were lysed using 15 strokes in a

7.0 ml Dounce homogenizer with pestle B. Cellular debris were cleared by

centrifugation for 3 minutes at 3000 rpm. To allow assessment of the INPUT for

HIV-1 p24, 100 μl of the cleared lysate were collected, made 1x in SDS sample

buffer, and analyzed by Western blotting. Then 2.0 ml of the cleared lysate were

layered onto a 50% sucrose (weight:volume) cushion in 1x PBS and centrifuged

at 125,000 x g for 2 hours at 4°C in a Beckman SW41 rotor. Following

centrifugation, 100 μl of the top-most portion of the supernatant were collected

and made 1x in SDS sample buffer; this sample is referred to as the Soluble

fraction. The Pellet was resuspended in 50 μl 1x SDS sample buffer and is

referred to as the particulate. All samples were then subjected to SDS-PAGE and

Western blotting. The HIV-1 p24 proteins were detected using a mouse anti-p24

antibody (Immuno Diagnostics). MxB proteins were detected using a rabbit anti-

FLAG antibody or anti-MxB antibodies (Novus Biologicals).

**MxB oligomerization assay**

Approximately 107 human 293 T cells were cotransfected with plasmids encoding

MxB variants tagged with FLAG and HA. After 24 h, cells were lysed in 0.5 ml of

whole-cell extract (WCE) buffer [50 mM Tris (pH 8.0), 280 mM NaCl, 0.5%

IGEPAL, 10% glycerol, 5 mM MgCl2, 50 μg/ml ethidium bromide, 50 U/ml

benzonase tail (Roche)]. Lysates were centrifuged at 14,000 rpm for 1 h at 4°C.

Post-spin lysates were then pre-cleared using protein A-agarose (Sigma) for 1 h

at 4°C; a small aliquot of each of these lysates was stored as input. Pre- cleared

lysates containing the tagged proteins were incubated with anti-FLAG-agarose

beads (Sigma) for 2 h at 4°C. Anti-FLAG- agarose beads were washed three

times in WCE buffer, and immune complexes were eluted using 200 mg of FLAG

tripeptide/ml in WCE buffer. The eluted samples were separated by SDS-PAGE

and analyzed by Western blotting using either anti-HA or anti-FLAG antibodies

(Sigma).

**Creation of cells stably expressing wild type and mutant MxB proteins**

Retroviral vectors encoding wild type or mutant human MxB proteins were

created using the LPCX vector. The MxB proteins contained either an influenza

hemagglutinin (HA) epitope tag or a FLAG epitope tag at the C terminus.

Recombinant viruses were produced in 293T cells by cotransfecting the LPCX

plasmids with the pVPack-GP and pVPack-VSV-G packaging plasmids

(Stratagene). The pVPack-VSV-G plasmid encodes the vesicular stomatitis virus

(VSV) G envelope glycoprotein, allowing efficient entry into a wide range of

vertebrate cells. HeLa, U937 and Cf2Th canine thymocytes were transduced and

selected in puromycin (Sigma).

**Western blot analysis**

Detection of proteins by Western blotting was performed using anti-FLAG

(Sigma), anti-HA (Sigma), anti-GAPDH (Ambion) or anti-p24 (Immuno

Diagnostics) antibodies. Secondary antibodies against rabbit and mouse

conjugated to IRDye 680LT or IRDye 800CW were obtained from LI-COR. Bands

were detected and quantified by scanning blots using the LI-COR Odyssey

Imaging System in the 700nm or 800nm channel.

**Mutagenesis**

The pET11a plasmids expressing the HIV-1 CA-NC containing the different

mutations were created by site-directed mutagenesis and confirmed by

sequencing analysis.

**HIV-1 CA–NC expression and purification**

The HIV-1 CA–NC protein was expressed, purified and assembled as previously

described(Ganser et al., 1999; Ganser-Pornillos et al., 2004). The pET11a

expression vector (Novagen) expressing the CA–NC protein of HIV-1 was used

to transform BL-21 (DE3) *Escherichia coli.* CA–NC expression was induced with

1 mM isopropyl-β-d-thiogalactopyranoside (IPTG) when the culture reached an

optical density of 0.6 at 600 nm. After 4 h of induction, cells were harvested and

resuspended in 20 mM Tris–HCl (pH 7.5), 500 mM NaCl, 1 μM ZnCl2, 10 mM 2-

mercaptoethanol and protease inhibitors (Roche). Lysis was performed by

sonication and debris was pelleted for 30 min at 35,000*g*. Nucleic acids were

stripped from the solution by using 0.11 equivalents of 2 M (NH4)2SO4 and the

same volume of 10% polyethylenimine. Nucleic acids were removed by stirring

and centrifugation at 29,500*g* for 15 min. Protein was recovered by addition of

0.35 equivalents of saturated (NH4)2SO4. The protein was centrifuged at 9820*g*

for 15 min and resuspended in 100 mM NaCl, 20 mM Tris–HCl (pH 7.5), 1 μM

ZnCl2 and 10 mM 2-mercaptoethanol. The CA–NC protein was dialyzed against

50 mM NaCl, 20 mM Tris–HCl (pH 7.5), 1 μM ZnCl2 and 10 mM 2-

mercaptoethanol, and stored at −80 °C.

**In vitro assembly of CA–NC complexes**

HIV-1 CA–NC particles were assembled in vitro by diluting the CA–NC protein to

a concentration of 0.3 mM in 50 mM Tris–HCl (pH 8.0), 0.5 M NaCl and 2 mg/ml DNA oligo-(TG)50. The mixture was incubated at 4 °C overnight and centrifuged

at 8600*g* for 5 min. The pellet was resuspended in assembly buffer (50 mM Tris–

HCl (pH 8.0), 0.5 M NaCl) at a final protein concentration of 0.15 mM (Ganser et

al., 1999; Ganser-Pornillos et al., 2004; Stremlau et al., 2006; Yang et al., 2014),

and stored at 4 °C until needed.

**Immunofluorescence microscopy**

Transfections of cell monolayers were performed using Lipofectamine Plus

reagent (Invitrogen), according to the manufacturer**’**s instructions. Transfections

were incubated at 37°C for 24 h. Indirect immunofluorescence microscopy was

perfomed as previously described (Brandariz-Nuñez et al., 2012). Transfected

monolayers grown on coverslips were washed twice with PBS1X (137 mM NaCl,

KCl 2.7 mM, Na2HPO4 . 2H2O 10 mM, KH2PO4 mM) and fixed for 15 min in

3.9% paraformaldehyde in PBS1X. Fixed cells were washed twice in PBS1X,

permeabilized for 4 min in permeabilizing buffer (0.5% Triton X-100 in PBS), and

then blocked in PBS1X containing 2% bovine serum albumin (blocking buffer) for

1 h at room temperature. Cells were then incubated for 1 h at room temperature

with primary antibodies diluted in blocking buffer. After three washes with PBS,

cells were incubated for 30 min in secondary antibodies and 1 mg of DAPI (49,

69-diamidino-2-phenylindole)/ml. Samples were mounted for fluorescence

microscopy by using the ProLong Antifade Kit (Molecular Probes, Eugene, OR).

Images were obtained with a ZeissObserver Z1 microscope using a 63x

objective, and deconvolution was performed using the software AxioVision V4.8.1.0 (Carl Zeiss Imaging Solutions).

**Virus production and Antibodies for co-localization experiments**

GFP Vpr/S15-mCherry fluorescently labeled R7ΔEnv HIV-1 virions pseudotyped

with VSV-g was generated as described before (Campbell et al., 2007). VSV-g

pseudotyped R7ΔEnv virions was generated in 10 cm plates of 293T cells with 7

μg R7ΔEnv GFP and 3 μg VSV-g using polyethylenimine (PEI). MX2 in the

stable cells is stained using Rabbit polyclonal Flag Ab (Thermo Scientific; PA1-

984B) and HIV capsid is stained using anti-p24 antibodies (clone AG3.0).

**Microscopy and data analysis**

Z-stack images were collected with a DeltaVision microscope equipped with a

digital camera using a 1.4-numerical aperture (NA) 100× objective lens, and were

deconvolved with SoftWoRx deconvolution software. Deconvolved images were

gated for their GFP-Vpr or p24 maximum fluorescence intensity by using the

surface finder function in the Imaris software (Bitplane). Fused virions were

determined as described before (Campbell et al., 2007).

**Virion-associated Reverse Transcriptase(RT)**

Virion-associated RT was quantified using an RT assay (Roche Applied Science,

Indianapolis, IN). To calculate the infectious units/ng RT of the different HIV-1 CA

mutant viruses, an amount of virus corresponding to an infectivity of less than

50% (M.O.I. <1) was used.
